# Oxidative stress-induced mitophagy is suppressed by the miR-106b-93-25 cluster in a protective manner

**DOI:** 10.1038/s41419-021-03484-3

**Published:** 2021-02-24

**Authors:** Cheng Zhang, Pengqing Nie, Chunliu Zhou, Yue Hu, Suling Duan, Meijia Gu, Dongxu Jiang, Yunfu Wang, Zixin Deng, Jincao Chen, Shi Chen, Lianrong Wang

**Affiliations:** 1 Key Laboratory of Combinatorial Biosynthesis and Drug Discovery, Ministry of Education, School of Pharmaceutical Sciences, Wuhan University, Wuhan, 430071 Hubei China; 2 Taihe Hospital, Hubei University of Medicine, Shiyan, 442000 Hubei China; 3 Brain Center, Department of Neurosurgery, Zhongnan Hospital, Wuhan University, Wuhan, 430071 Hubei China; 4 Department of Burn and Plastic Surgery, Division of Wound Repair, Shenzhen Institute of Translational Medicine, the First Affiliated Hospital, Shenzhen University, Shenzhen, 518035 Guangdong China

**Keywords:** Mitophagy, miRNAs

## Abstract

Increased reactive oxygen species levels in the mitochondrial matrix can induce Parkin-dependent mitophagy, which selectively degrades dysfunctional mitochondria via the autolysosome pathway. Phosphorylated mitofusin-2 (MFN2), a receptor of parkin RBR E3 ubiquitin-protein ligase (Parkin), interacts with Parkin to promote the ubiquitination of mitochondrial proteins; meanwhile, the mitophagy receptors Optineurin (OPTN) and nuclear dot protein 52 (NDP52) are recruited to damaged mitochondria to promote mitophagy. However, previous studies have not investigated changes in the levels of OPTN, MFN2, and NDP52 during Parkin-mediated mitophagy. Here, we show that mild and sustained hydrogen peroxide (H_2_O_2_) stimulation induces Parkin-dependent mitophagy accompanied by downregulation of the mitophagy-associated proteins OPTN, NDP52, and MFN2. We further demonstrate that H_2_O_2_ promotes the expression of the miR-106b-93-25 cluster and that miR-106b and miR-93 synergistically inhibit the translation of OPTN, NDP52, and MFN2 by targeting their 3’ untranslated regions. We further reveal that compromised phosphorylation of MYC proto-oncogene protein (c-Myc) at threonine 58 (T58) (producing an unstable form of c-Myc) caused by reduced nuclear glycogen synthase kinase-3 beta (GSK3β) levels contributes to the promotion of miR-106b-93-25 cluster expression upon H_2_O_2_ induction. Furthermore, miR-106b-mediated and miR-93-mediated inhibition of mitophagy-associated proteins (OPTN, MFN2, and NDP52) restrains cell death by controlling excessive mitophagy. Our data suggest that microRNAs (miRNAs) targeting mitophagy-associated proteins maintain cell survival, which is a novel mechanism of mitophagy control. Thus, our findings provide mechanistic insight into how miRNA-mediated regulation alters the biological process of mitophagy.

## Introduction

Impaired mitochondria can be discriminated from normal mitochondria and cleared by the autophagosome-lysosome pathway^1^. One common mechanism for mitophagy involves the PTEN-induced kinase 1 (PINK1)/parkin RBR E3 ubiquitin-protein ligase (Parkin) pathway. When mitochondria are damaged, PINK1 is stabilized and phosphorylates mitofusin-2 (MFN2); phosphorylated MFN2 then recruits Parkin, which links to polyubiquitin, to dysfunctional mitochondria^2^. TBK1-phosphorylated Optineurin (OPTN) and nuclear dot protein 52 (NDP52) interact with polyubiquitin chains through ubiquitin-binding domains, and the microtubule-associated protein 1 light chain 3 (LC3)-interacting regions (LIRs) of OPTN and NDP52 then recruit LC3 along with autolysosomes to clear damaged mitochondria^3–5^. Numerous reports have suggested that disordered mitochondrial dynamics, disruption of mitochondrial function, and mutations in mitophagy receptors/adapters contribute to neurodegenerative diseases^6–8^.

Mitochondrial respiratory chain inhibitors and uncoupling agents are frequently used to induce mitophagy^9^. However, none of these inhibitors or reagents are endogenous molecules. Reactive oxygen species (ROS), which are endogenous and pervasive in cells, can cause loss of mitochondrial membrane potential, thus activating Parkin-dependent mitophagy^10,11^. Notably, ROS has been reported to act as the second messengers in cell signaling^12,13^. We sought to investigate whether ROS-mediated signal transduction and mitophagy regulation correlate.

MicroRNAs (miRNAs), a class of endogenous short noncoding RNAs composed of ~23 nucleotides, regulate genes by binding to the 3’ untranslated regions (3’UTRs) of targeted mRNAs, resulting in translation inhibition and mRNA degradation^14^. Previous studies indicate that miRNAs participate in the regulation of mitophagy. miR-137, a novel hypoxia-responsive miRNA, can suppress the expression of FUNDC1 and NIX to inhibit mitophagy^15^. miR-27a and miR-27b inhibit PINK1 expression by binding to the 3’UTR of PINK1 mRNA to regulate mitophagy^16^.

Frank and Wang have suggested that ROS at high concentrations specifically induce PINK1/Parkin-dependent mitophagy^10,17^. However, high concentrations of H_2_O_2_ can quickly cause cell death^18^. Therefore, we exposed cells to relatively low concentrations of H_2_O_2_ for extended durations to simulate ROS elevation in vivo. In this study, we found that mild and sustained stimulation with H_2_O_2_ modulated mitochondrial morphology and induced Parkin-mediated mitophagy. In parallel, by acting as a signaling molecule, H_2_O_2_ regulated the GSK3β/c-Myc pathway, promoting the expression of the miR-106b-93-25 cluster to inhibit the mitophagy-associated proteins OPTN, NDP52, and MFN2 by binding to the 3’UTRs of their mRNAs. Finally, we demonstrated that miRNAs inhibition of OPTN, NDP52, and MFN2 genes can maintain cell survival by controlling excessive mitophagy.

## Results

### Mild and sustained stimulation with H_2_O_2_ modulates mitochondrial morphology and induces Parkin-mediated mitophagy

**Fig. 1:**
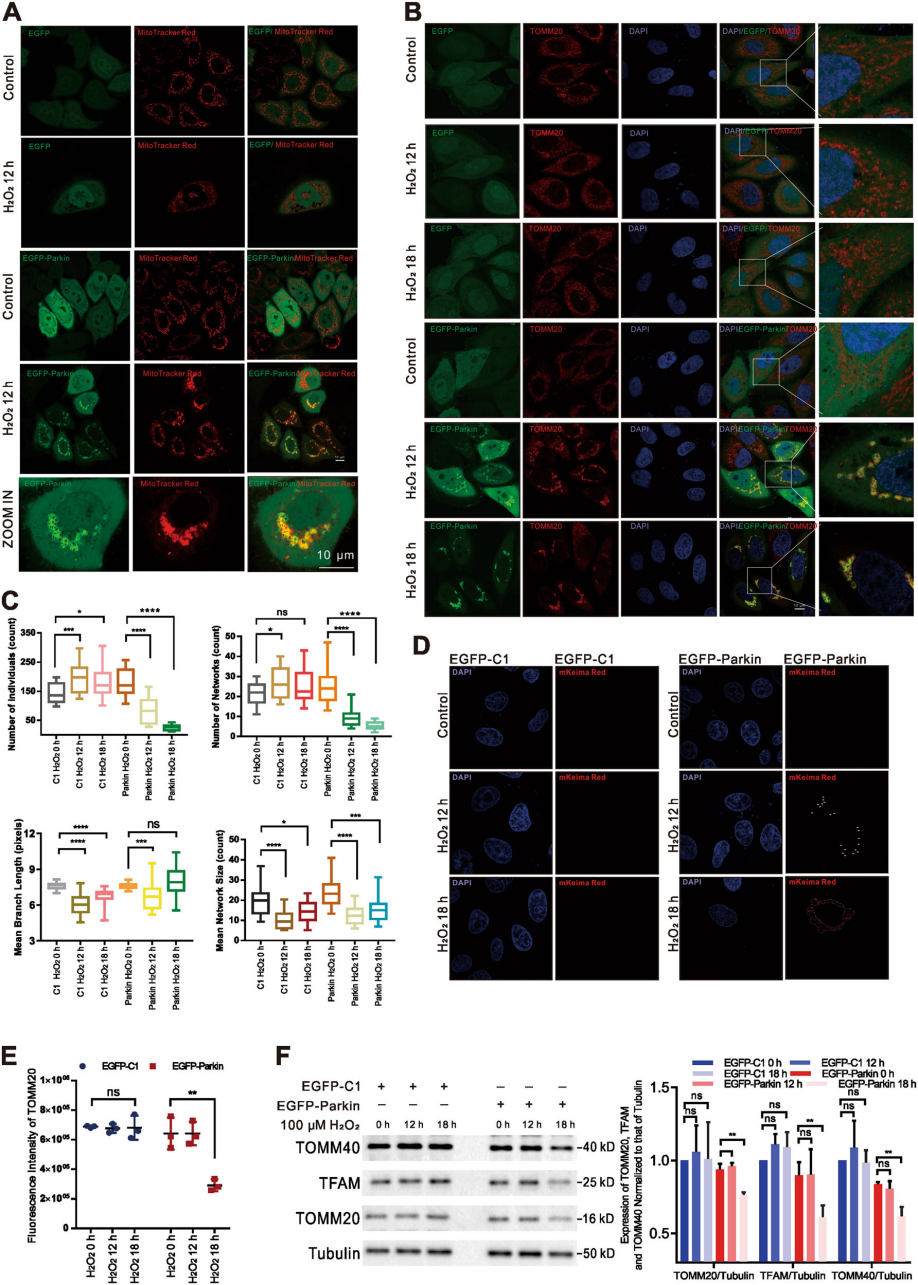
Mild and sustained stimulation with H_2_O_2_ modulates mitochondrial morphology and induces Parkin-mediated mitophagy. **A** EGFP-C1 (as a control) or EGFP-Parkin were transfected into cells before 0 h and 12 h of H_2_O_2_ treatment. Mitochondria were stained with MitoTracker Red (red). Scale bars, 10 μm. **B** Cells transfected with EGFP-C1 or EGFP-Parkin were incubated with 100 μΜ H_2_O_2_ for 0 h, 12 h, or 18 h. The cells were immunostained with an anti-TOMM20 antibody (red). Scale bars, 10 μm. **C** Mitochondrial morphology was quantitatively analyzed using the MiNA ImageJ macro tool based on fluorescent images of TOMM20 (~20 cells for each analysis). Unpaired *t-*test; ns, not significant; *P < 0.05; ***P < 0.001; ****P < 0.0001. The data are presented as the mean ± SD. **D** HeLa cells were transfected with the mKeima-Red-Mito-7 plasmid along with EGFP-Parkin or EGFP-C1 (as a control) and then treated with 100 μΜ H_2_O_2_ for 0 h, 12 h, or 18 h. The white arrows and dashed box show the red fluorescent dots of Keima excited at 560 nm. Scale bar, 10 μm. **E** The fluorescence intensity of TOMM20 was analyzed by ImageJ in three independent experiments, and the results were subjected to statistical analysis. Ns, not significant; **P < 0.01. **F** Cells were transfected with EGFP-C1 or EGFP-Parkin plasmids and treated with 100 μΜ H_2_O_2_ for 0 h, 12 h, or 18 h. The levels of the mitochondrial markers TFAM, TOMM40, and TOMM20 were evaluated by WB analysis. *N* = 3; ns, not significant; **P < 0.01. The quantified results are presented as the mean ± SD.

Accumulation of ROS can cause loss of mitochondrial membrane potential and changes in mitochondrial permeability following disruption of mitochondrial dynamics^10,17,19,20^, suggesting that ROS are distinct inducers of mitophagy; nevertheless, the induction of high concentrations of ROS is extremely unfavorable for cells, and acute mitophagy masks many details of cell self-regulation. We investigated the occurrence of mitophagy and the effects of mitophagy induced by mild (low-concentration) and sustained ROS exposure on mitochondrial morphology and function. HeLa cells were stimulated with 100 μM H_2_O_2_ for different lengths of time. HeLa cells do not express Parkin^21^; thus, EGFP-Parkin was transfected into these cells to supplement them with Parkin, and EGFP-C1 was transfected as a control (Supplementary Fig. 1A). Using MitoTracker Red to label mitochondria, we observed that mitochondrial morphology changed after 12 h of H_2_O_2_ stimulation in cells expressing both EGFP-Parkin and EGFP-C1; the Parkin was recruited to mitochondria (Fig. 1A). Translocase of outer mitochondrial membrane 20 (TOMM20), located on the mitochondrial outer membrane, was used to label mitochondria for immunofluorescence (IF). In EGFP-C1-transfected cells, the number of individual mitochondria increased, while the mean branch length and mean network size decreased dramatically (Fig. 1B, C), indicating that mitochondrial fragmentation occurred after 12–18 h of H_2_O_2_ stimulation. In EGFP-Parkin-transfected cells, the number of individual and network mitochondria decreased in a time-dependent manner, and the mean network size was reduced; these results showed that mitochondria aggregated after 12–18 h of H_2_O_2_ stimulation (Fig. 1B, C). These data revealed that mitochondria with normal morphology transformed into tight clusters and that Parkin was recruited to the clustered mitochondria. In the absence of Parkin, mitochondria became fragmented but did not aggregate. A similar change in mitochondrial morphology occurred after 12 h of H_2_O_2_ stimulation in the CFTF cell line (Supplementary Results). Together, these results suggest that mild and sustained H_2_O_2_ stimulation can induce a change in mitochondrial morphology, and this change is upstream of Parkin translocation to mitochondria.

Parkin recruitment to mitochondria is a trigger for Parkin-mediated mitophagy. Hence, we cotransfected HeLa cells with mKeima-Red-Mito-7 and EGFP-Parkin or EGFP-C1 to observe whether mitophagy was induced by H_2_O_2_ stimulation. mKeima-Red-Mito-7 expresses the pH-sensitive fluorescent protein Keima fused with a mitochondria-localizing peptide. Keima emits green fluorescence in a neutral environment but fluoresces red under acidic lysosomal conditions. As shown in Fig. 1D, among EGFP-Parkin-transfected cells, a few red dots appeared in the cytoplasm (white arrows) after 12 h of H_2_O_2_ treatment, and many red dots appeared (dashed box) after 18 h of H_2_O_2_ treatment. In contrast, no red dots appeared in EGFP-C1-transfected cells. These results indicated that H_2_O_2_ induced mitochondria to form autolysosomes in the presence of Parkin. In addition, H_2_O_2_ stimulation for 18 h decreased TOMM20 fluorescence intensity in EGFP-Parkin-transfected cells, whereas H_2_O_2_ rarely changed TOMM20 fluorescence intensity in EGFP-C1-transfected cells (Fig. 1E). Fluorescence intensity of transcription factor A, mitochondrial (TFAM) was also significantly decreased in EGFP-Parkin-transfected cells after 18 h of H_2_O_2_-stimulation but was unchanged in EGFP-C1-transfected cells (Supplementary Fig. 3A). Furthermore, we observed decreased translocase of outer mitochondrial membrane 40 (TOMM40), TOMM20, and TFAM levels when EGFP-Parkin-transfected cells were induced with H_2_O_2_ for 18 h, but H_2_O_2_ treatment had no effect on the expression of these proteins in the absence of Parkin (Fig. 1F). For further confirmation, we stably expressed EGFP (as a control) and EGFP-Parkin in HeLa cells with a lentiviral system and named these the EGFP-Control cell line and the EGFP-Parkin cell line (Supplementary Fig. 1D). Decreased TOMM40 and TFAM levels were observed in EGFP-Parkin cell lines induced with H_2_O_2_ for 18 h (Supplementary Fig. 3B). These observations indicate that mild and sustained stimulation with H_2_O_2_ can induce mitophagy. Moreover, H_2_O_2_-induced mitophagy occurs in a Parkin-dependent manner.

### H_2_O_2_ induction modulates mitophagy-associated protein levels

**Fig. 2:**
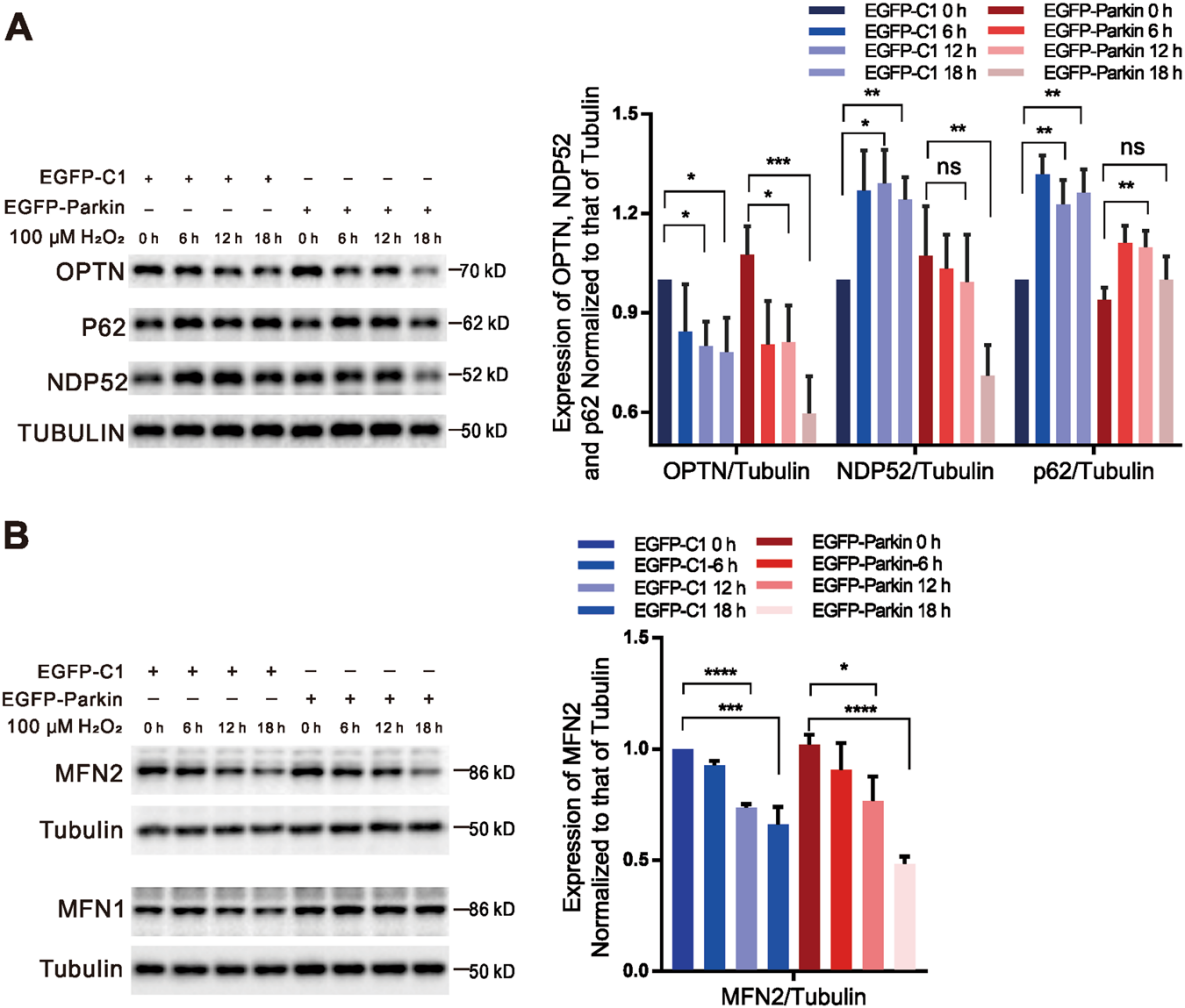
H_2_O_2_ induction modulates mitophagy-associated protein levels. **A** Cells were transfected with the EGFP-C1 (as a control) or EGFP-Parkin plasmids. Twenty-four hours later, the cells were treated with 100 μΜ H_2_O_2_ for 0 h, 6 h, 12 h, or 18 h. The levels of the mitophagy-associated proteins OPTN, NDP52, and p62 were evaluated by WB analysis. *N* = 4; ns, not significant; *P < 0.05; **P < 0.01; ***P < 0.001. Data from four independent tests were collected for statistical analysis (mean ± SD). **B** Cells were transfected with the EGFP-C1 or EGFP-Parkin plasmids. Twenty-four hours later, the cells were treated with 100 μΜ H_2_O_2_ for 0 h, 6 h, 12 h, or 18 h. The levels of MFN2 and MFN1 were evaluated by WB analysis. *N* = 3; *P < 0.05; ***P < 0.001; ****P < 0.0001. Data from three independent tests were collected for statistical analysis (mean ± SD).

OPTN and NDP52 are the primary, yet redundant receptors involved in Parkin-mediated mitophagy, while p62 and NBR1 are dispensable for Parkin-mediated mitophagy^5^. p62 levels are increased after H_2_O_2_-treatment and have neuroprotective effects against H_2_O_2_-induced cell death^22^. In this study, OPTN levels were decreased in both EGFP-Parkin-transfected and EGFP-C1-transfected cells after 12–18 h of H_2_O_2_ stimulation, while NDP52 levels were decreased only in EGFP-Parkin-transfected cells after 18 h of H_2_O_2_ stimulation (Fig. 2A). In cells not expressing Parkin, p62 levels increased upon H_2_O_2_-induction. However, p62 levels increased after 6–12 h of H_2_O_2_-induction and then declined after 18 h in EGFP-Parkin-transfected cells (Fig. 2A). The strong induction of p62 by H_2_O_2_ implied that the cells were under oxidative stress^23^. After H_2_O_2_-induction for 18 h, p62 levels in EGFP-Parkin-transfected cells returned to normal, which suggested that oxidative stress was relieved via Parkin-mediated mitophagy. We propose that cells acclimatize to oxidative stress by eliminating damaged mitochondria.

PINK1-phosphorylated MFN2 recruits Parkin to promote its ubiquitination to drive mitophagy in a p97-dependent manner^2,24^. We found that MFN2 levels declined in both EGFP-Parkin-transfected and control cells after 12–18 h of H_2_O_2_ treatment (Fig. 2B). In addition, the levels of mitofusin 1 (MFN1) participating in mitochondrial fusion together with MFN2, changed little (Fig. 2B). Consistent with the results shown in Fig. 2A and B, OPTN, NDP52, and MFN2 levels declined in EGFP-Parkin cell lines after 18 h of H_2_O_2_ treatment (Supplementary Fig. 4). We speculate that changes in mitophagy-associated proteins have positive effects on cells, but why these proteins change requires further investigation.

### miR-106b and miR-93 synergistically suppress the expression of OPTN, NDP52, and MFN2

**Fig. 3:**
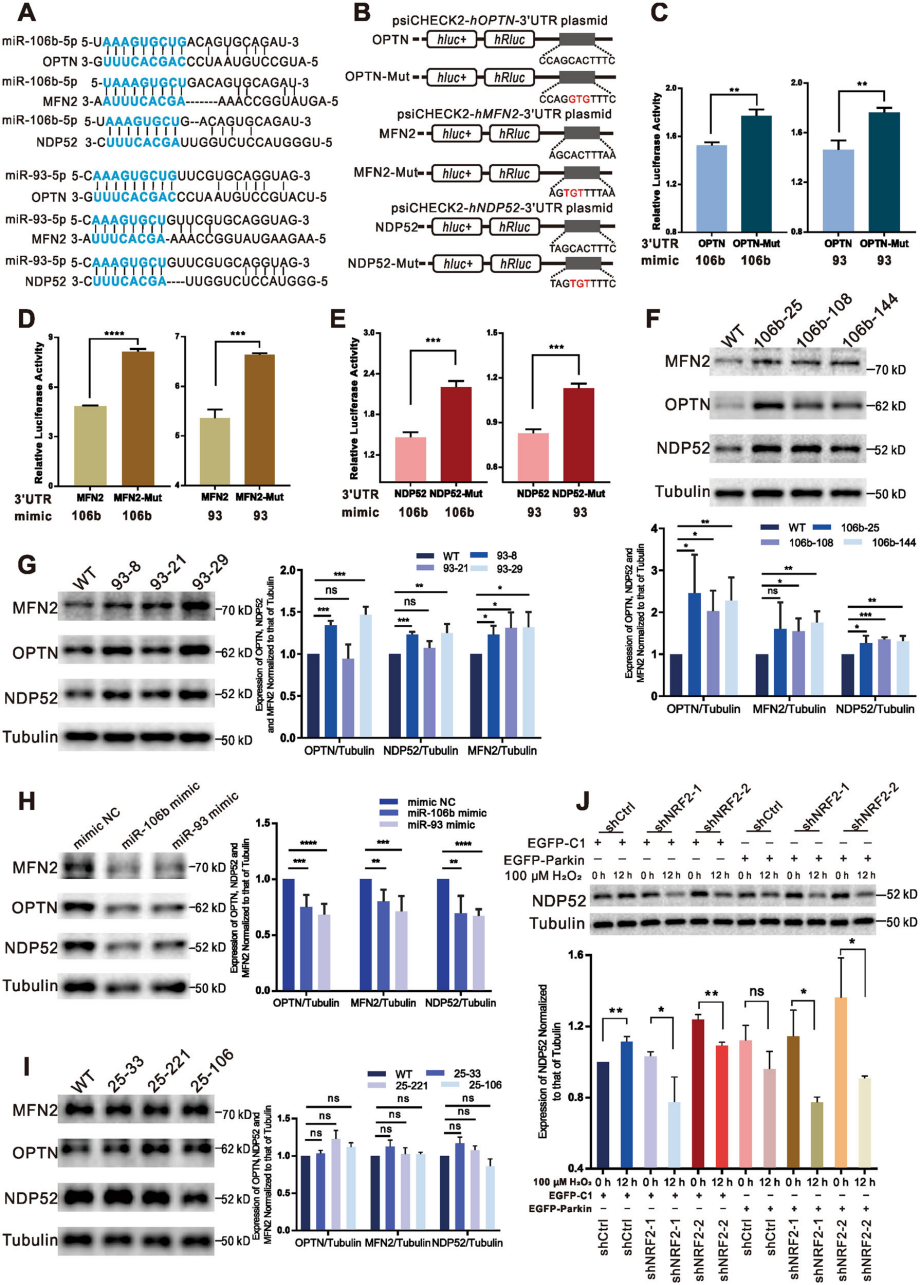
miR-106b and miR-93 synergistically suppress the expression of OPTN, NDP52, and MFN2. **A** Schematic showing the sequences of human miR-106b and miR-93, which were predicted to target the 3’UTRs of OPTN, NDP52, and MFN2. **B** Schematics representing the reconstructed dual-luciferase reporter plasmids. The WT 3’UTRs of OPTN, NDP52, and MFN2 containing the miRNA-binding sequences were cloned downstream of Renilla luciferase (*hRluc*). Artificially mutated miRNA-binding sequences (OPTN-Mut, MFN2-Mut, and NDP52-Mut) were also cloned downstream of Renilla luciferase. Mutations are marked in red. **C** Cells were transfected with reporter plasmids containing the WT 3’UTR of OPTN or a mutant OPTN 3’UTR (OPTN-Mut) along with a miR-106b mimic or miR-93 mimic for 48 h. Firefly luciferase (*hluc*+) and Renilla luciferase (*hRluc*) activity levels were measured successively, and the values are presented as the relative fluorescence intensity (*hRluc/hluc*+). *N* = 3; **P < 0.01. The quantified results are presented as the mean ± SD. **D** Cells were transfected with reporter plasmids containing the WT 3’UTR of MFN2 or a mutant MFN2 3’UTR (MFN2-Mut) along with a miR-106b mimic or miR-93 mimic for 48 h. The Renilla luciferase activity was normalized to the firefly luciferase activity. *N* = 3; ***P < 0.005; ****P < 0.001. The quantified results are presented as the mean ± SD. **E** Cells were transfected with reporter plasmids containing the WT 3’UTR of NDP52 or a mutant NDP52 3’UTR (NDP52-Mut) along with a miR-106b mimic or miR-93 mimic for 48 h. The normalized luciferase activity (ratio of *hRluc* to *hluc*+) was measured in cell lysates. *N* = 3; ***P < 0.005. The quantified results are presented as the mean ± SD. **F**, **G**, **I** The levels of OPTN, NDP52, and MFN2 in WT cells, three miR-106b-KO cell lines, three miR-93-KO cell lines, and three miR-25-KO cell lines were evaluated by WB analysis. *N* = 3; ns, not significant; *P < 0.05; **P < 0.01; ***P < 0.001. The data are from three independent tests and are presented as the mean ± SD. **H** WB analysis of OPTN, NDP52, and MFN2 levels 48 h after transfection with a miRNA mimic NC, a miR-106b mimic, or a miR-93 mimic. Tubulin was used as an endogenous control. *N* = 3; **P < 0.01; ***P < 0.001; ****P < 0.0001. The data are from three independent tests and are presented as the mean ± SD. **J** Three cell lines (ShCtrl, shRNF2-1, and shRNF2-2) were transfected with EGFP-C1 or EGFP-Parkin and stimulated with 100 μΜ H_2_O_2_ for 0 h or 12 h. The cell lysates were subjected to WB analysis using the indicated antibodies. *N* = 3; ns, not significant; *P < 0.05; **P < 0.01. The data are from three independent tests and are presented as the mean ± SD.

OPTN, NDP52, and MFN2 are involved in Parkin-mediated mitophagy^2,4,5^. By analyzing discordant changes in mRNA and protein levels of OPTN, NDP52, and MFN1, and concordant changes in MFN2 mRNA and protein, we demonstrate that OPTN, NDP52, and MFN2 translation is inhibited (Supplementary Results). miRNAs play an extensive role in posttranslational regulation, including translation inhibition and mRNA degradation^14^. Using the miRDB and TargetScanHuman miRNA target-predicting websites, we identified two miRNAs (miR-106b and miR-93) that were predicted to target OPTN, NDP52, and MFN2. miR-106b, miR-93, and miR-25 are encoded by the polycistronic miR-106b-93-25 cluster, which is embedded in intron 13 of the *MCM7* gene. Sequence alignment showed that miR-106b and miR-93 can bind to the 3’UTRs of OPTN, NDP52, and MFN2 (Fig. 3A). Levels of key miRNAs are upregulated or downregulated, which results in altered expression of target proteins^14^. Hence, we speculate that the levels of miR-106b and miR-93 might be elevated upon H_2_O_2_-induction. Because OPTN, MFN2, and NDP52 levels were decreased in EGFP-Parkin-transfected cells and OPTN and MFN2 levels were decreased in EGFP-C1-transfected cells after H_2_O_2_ treatment, we measured levels of miR-106b, miR-93, and miR-25, which are transcribed simultaneously as a miRNA cluster, in both groups (cells transfected with EGFP-Parkin and EGFP-C1). As expected, miR-106b, miR-93, and miR-25 levels were increased upon 12 h of H_2_O_2_ induction in both groups (Supplementary Fig. 5B). This increased expression of miR-106b and miR-93 correlated with the downregulation of these proteins, suggesting that miR-106b and miR-93 regulate OPTN, MFN2, and NDP52 regardless of whether Parkin is present.

To validate the direct effects of the miRNAs on these proteins, the wild-type (WT) 3’UTRs of OPTN, NDP52, and MFN2 containing the miRNA-binding sequences were cloned downstream of Renilla luciferase in psiCHECK-2 (Fig. 3B). In parallel, constructs with three consecutive base mutations in the miRNA-binding sites were created as negative controls (Fig. 3B). Cotransfection of one miRNA mimic (miR-106b or miR-93) together with the WT psiCHECK2-h*OPTN*-3’UTR plasmid into HeLa cells led to a significant decrease in relative luciferase activity (*hRluc/hluc*+) compared with the level in the negative control cells (transfected with OPTN-Mut) (Fig. 3C). Similarly, miR-106b and miR-93 significantly suppressed the activity of Renilla luciferase in cells with the WT 3’UTRs of MFN2 and NDP52 compared with cells with the negative control 3’UTRs (MFN2-Mut and NDP52-Mut) (Fig. 3D, E). Collectively, these data indicate that miR-106b and miR-93 suppress OPTN, MFN2, and NDP52 expression by directly targeting the 3’UTRs of their mRNA.

To confirm the results, we generated miR-106b-knockout (KO), miR-93-KO, and miR-25-KO HeLa cells using CRISPR/Cas9 (Supplementary Fig. 6A–D). As shown by the western blot (WB) results in Fig. 3F, the levels of OPTN, NDP52, and MFN2 were increased in each miR-106b-KO cell line. Furthermore, we found that miR-106b suppressed these proteins to different degrees. OPTN was regulated to the greatest extent, followed by MFN2 and NDP52. To verify this result, OPTN, NDP52, and MFN2 levels in HeLa cells transfected with miR-106b mimic or mimic negative control (mimic NC) were assessed by WB analysis. The levels of OPTN, NDP52, and MFN2 were distinctly decreased in cells transfected with miR-106b mimic (Fig. 3H). MFN2 levels were increased in all miR-93-KO cell lines, while OPTN and NDP52 levels were increased in all miR-93-KO cell lines, except for 93-21 (Fig. 3G). In addition, the levels of OPTN, NDP52, and MFN2 were significantly decreased in the miR-93-mimic-transfected cells (Fig. 3H). These results showed that miR-93 was the main regulator of MFN2 and an auxiliary regulator of OPTN and NDP52. Moreover, levels of OPTN, NDP52, and MFN2 in miR-25-KO cells (25-33, 25-221, and 25-106) were not obviously different from those in WT cells (Fig. 3I). Altogether, these results suggest that miR-106b and miR-93 synergistically suppress OPTN, NDP52, and MFN2 to varying degrees.

Although NDP52 is downregulated by miR-106b and miR-93, NDP52 levels did not decrease after 12 h of H_2_O_2_ treatment (Fig. 2A). H_2_O_2_ stimulation in HeLa cells can increase intracellular ROS levels (Supplementary Fig. 7A). When cells undergo oxidative stress, the Nrf2-Keap1 pathway is activated, after which NRF2 translocates into the nucleus to bind to the NDP52 promoter and induce NDP52 expression^25,26^. We hypothesized that NRF2-promoted effects on NDP52 neutralize the suppression of NDP52 by miR-106b and miR-93. Using a lentivirus system, we generated two NRF2-knockdown cell lines (shNRF2-1 and shNRF2-2) stably expressing short hairpin RNA (shRNA) targeting NRF2 and one control cell line (shCtrl) expressing scrambled shRNA (Supplementary Fig. 1E). Base on IF, nuclear NRF2 levels increased significantly after 12 h of H_2_O_2_ treatment (Supplementary Fig. 7B). In EGFP-C1-transfected cells, H_2_O_2_ stimulation for 12 h reduced NDP52 levels in NRF2-knockdown cell lines but enhanced NDP52 expression in the shCtrl cell line (Fig. 3J). Among EGFP-Parkin-expressing cells, NDP52 levels were decreased in NRF2-knockdown cell lines but unchanged in the shCtrl cell line after 12 h of H_2_O_2_ stimulation (Fig. 3J). These findings indicate that NRF2 knockdown reduces its capacity to regulate NDP52 and that increased levels of miR-106b and miR-93 induced by H_2_O_2_ cosuppress NDP52 expression.

### H_2_O_2_ stimulation promotes the expression of c-Myc by decreasing the level of GSK3β in the nucleus

**Fig. 4:**
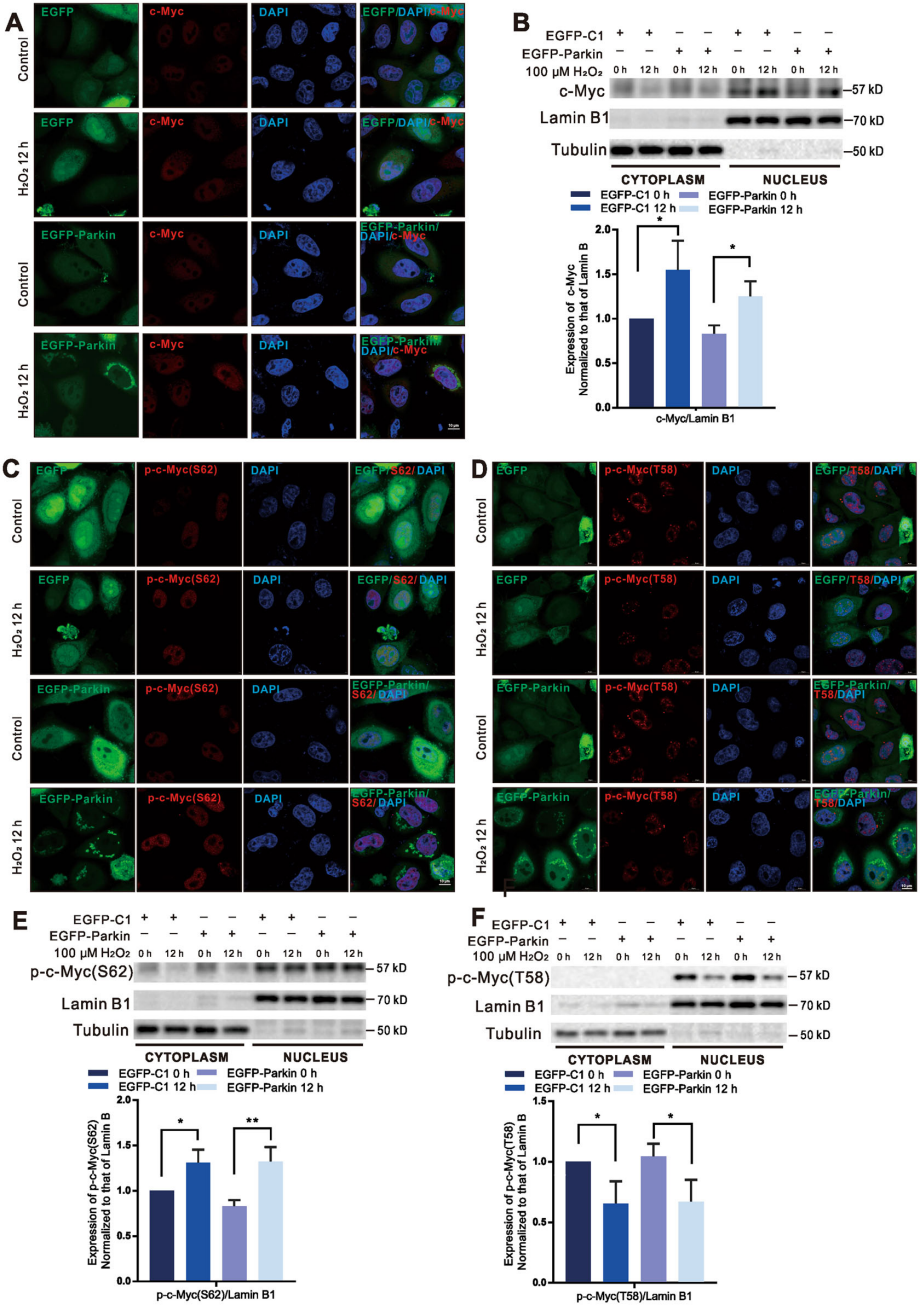
c-Myc is upregulated in H_2_O_2_-induced cells. **A, C, D** EGFP-Parkin-transfected and EGFP-C1-transfected HeLa cells were stimulated with 100 μΜ H_2_O_2_ for 0 h or 12 h and stained with anti-Myc, anti-Myc-S^62^, and anti-Myc-T^58^ antibodies (red). Scale bars, 10 μm. **B**, **E**, **F** Cells were transfected with EGFP-C1 or EGFP-Parkin plasmids. After 24 h, the cells were treated with 100 μΜ H_2_O_2_ for 0 h or 12 h. Nuclear extracts were immunoblotted for c-Myc, p-c-Myc-S62, and p-c-Myc-T58. Lamin B1 was used as a nuclear internal reference, and Tubulin was used as a cytoplasmic internal reference. *N* = 3; *P < 0.05; **P < 0.01. The data are from three independent tests and are presented as the mean ± SD.

Previous studies have reported that the miR-106b-93-25 cluster is regulated by c-Myc^27–30^. In addition, MCM7, as the host gene of miR-106b-93-25, is upregulated by c-Myc^31,32^. The fluorescence intensity of c-Myc increased after the treatment of both EGFP-Parkin-transfected and EGFP-C1-transfected cells with H_2_O_2_ for 12 h (Fig. 4A). Furthermore, we isolated nuclei from whole-cell lysates for WB analysis. As expected, c-Myc levels increased after 12 h of H_2_O_2_ treatment (Fig. 4B). Posttranslational regulation of c-Myc involves a series of proteins that sequentially alter the phosphorylation states of two conserved residues, serine 62 (S62) and threonine 58 (T58); S62 phosphorylation stabilizes c-Myc, while T58 phosphorylation destabilizes c-Myc^33,34^. IF showed that the levels of phosphorylated c-Myc at S62 (p-c-Myc-S62) increased and those of phosphorylated c-Myc at T58 (p-c-Myc-T58) decreased after 12 h of H_2_O_2_ treatment in both EGFP-Parkin-expressing cells and EGFP-C1 control cells (Fig. 4C, D). Similarly, the WB results revealed that p-c-Myc-S62 levels increased and p-c-Myc-T58 levels decreased after H_2_O_2_ treatment (Fig. 4E, F). Taken together, these results suggest that H_2_O_2_ upregulates the miR-106b-93-25 cluster via the upregulation of c-Myc.

**Fig. 5:**
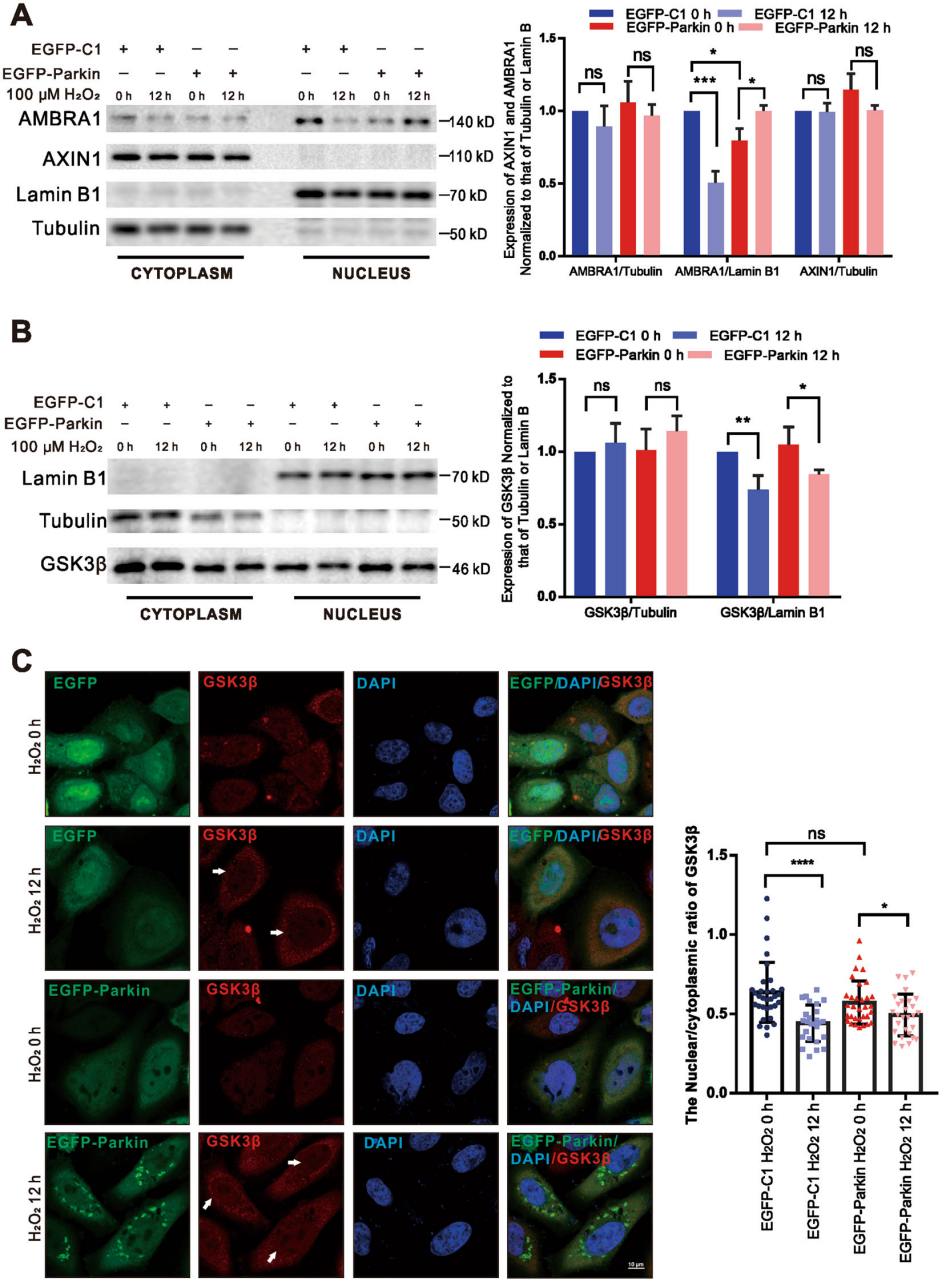
H_2_O_2_ stimulation promotes the expression of c-Myc by decreasing GSK3β levels in the nucleus. **A**, **B** Cells were transfected with EGFP-C1 or EGFP-Parkin and then stimulated with 100 μΜ H_2_O_2_ for 0 h or 12 h. Then, nuclear and cytoplasmic proteins were isolated for WB analysis. The protein levels were analyzed with the indicated antibodies. *N* = 3; ns, not significant; *P < 0.05; **P < 0.01; ***P < 0.001. The data are from three independent tests and are presented as the mean ± SD. **C** Cells transfected with EGFP-C1 (green) or EGFP-Parkin (green) were incubated with 100 μΜ H_2_O_2_ for 0 h or 12 h. The cells were immunostained with an anti-GSK3β antibody (red). The white arrows show GSK3β in the nucleus. The fluorescence intensity was analyzed with ImageJ, and the data were subjected to statistical analysis (~30 cells for each analysis). Scale bars, 10 μm. Ns, not significant; *P < 0.05; ****P < 0.0001.

PP2A-B56α, Pin1 and the scaffold protein Axin 1 cooperate to dephosphorylate c-Myc at S62 (ref. ^33,35^). In addition, AMBRA1 destabilizes c-Myc by enhancing the PP2A-induced c-Myc-S62 dephosphorylation^36^. In both EGFP-Parkin-transfected and EGFP-C1-transfected cells, the levels of AXIN1, AMBRA1, and PP2A in the cytoplasm did not noticeably change after H_2_O_2_ treatment; likewise, endonuclear PP2A did not obviously change, while endonuclear AMBRA1 changed irregularly (Fig. 5A and Supplementary Fig. 8A), indicating that dephosphorylation at S62 was not responsible for c-Myc degradation under H_2_O_2_ stress. GSK3β, a proline-directed serine-threonine kinase, phosphorylates c-Myc at T58 for c-Myc degradation^37^. Interestingly, GSK3β was significantly downregulated in cell nuclear lysates after H_2_O_2_-induction for 12 h in both EGFP-Parkin-transfected and EGFP-C1-transfected cells (Fig. 5B). IF also showed that GSK3β was partially degraded in the nuclei (Fig. 5C). Furthermore, the levels of endonuclear phosphorylated GSK3β at serine 9 (p-GSK3β-S9, an inactive form of GSK3β) were increased after 12 h of H_2_O_2_ treatment (Supplementary Fig. 8B). These results show that ROS, which acts as signaling molecules, reduce GSK3β levels in the nucleus to upregulate c-Myc.

### The miR-106b-93-25 cluster protects cells from excessive mitophagy

**Fig. 6:**
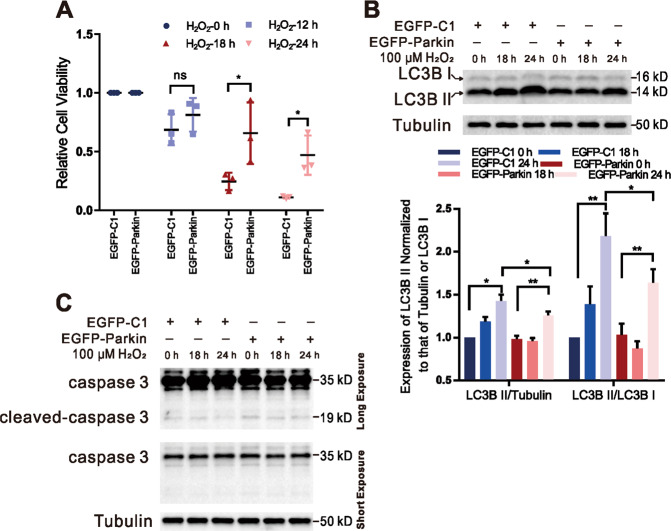
Parkin-mediated mitophagy restrains H_2_O_2_-induced cell death. **A** Cells were transfected with EGFP-C1 or EGFP-Parkin plasmids. Twenty-four hours later, cell viability was detected after H_2_O_2_ induction (100 μΜ; 0 h, 12 h, 18 h, or 24 h). *N* = 3; ns, not significant; *P < 0.05. Data from three independent tests were collected for statistical analysis (mean ± SD). **B** Cells were transfected with EGFP-C1 or EGFP-Parkin and then stimulated with 100 μΜ H_2_O_2_ for 0 h, 18 h, or 24 h. The levels of the autophagy marker LC3B were evaluated by WB analysis. Tubulin was used as an endogenous control. *N* = 3; *P < 0.05; **P < 0.01. The data are from three independent tests and are presented as the mean ± SD. **C** Cells were transfected with EGFP-C1 or EGFP-Parkin plasmids. Twenty-four hours later, the cells were treated with 100 μΜ H_2_O_2_ for 0 h, 18 h, or 24 h. The levels of caspase 3 and cleaved caspase 3 were evaluated by WB analysis. The upper band underwent a long exposure, and the lower band underwent a short exposure. Tubulin was used as an internal reference.

**Fig. 7:**
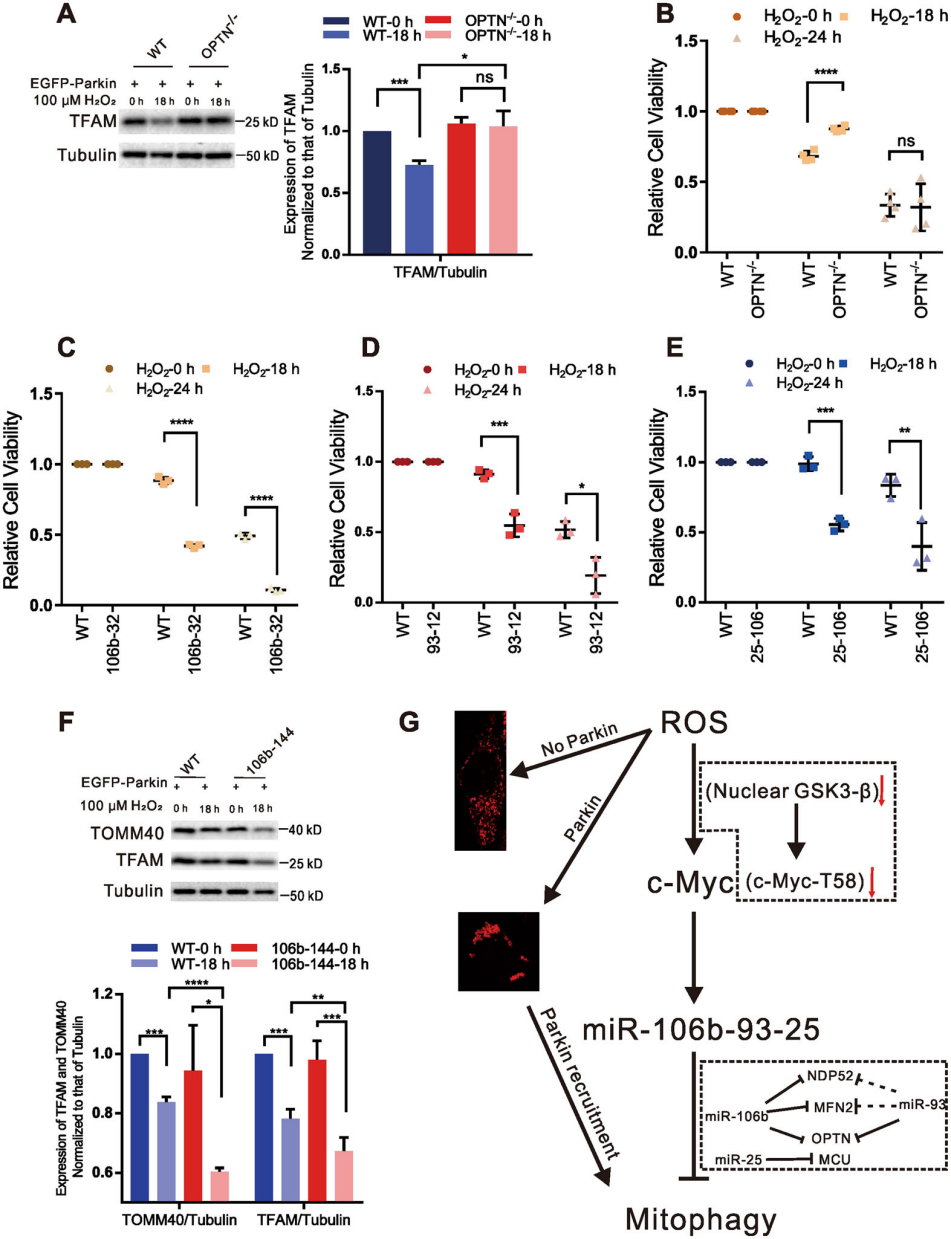
The miR-106b-93-25 cluster protects cells from excessive mitophagy. **A** EGFP-Parkin was transfected into WT and *OPTN-KO* HeLa cells for 24 h. WB analysis of TFAM was performed after 0–18 h of 100 μΜ H_2_O_2_ treatment. Tubulin was used as an endogenous control. *N* = 3; ns, not significant; *P < 0.05; ***P < 0.001. The data are from three independent tests and are presented as the mean ± SD. **B**–**E** A WT cell line, an *OPTN-KO* HeLa cell line, a miR-106b-KO cell line (106b-32), a miR-93-KO cell line (93-12), and a miR-25-KO cell line (25-106) were transfected with the EGFP-Parkin plasmid. Cell viability was detected after H_2_O_2_ induction (100 μΜ; 0 h, 18 h, or 24 h). *N* = 4 or 3; ns, not significant; *P < 0.05; **P < 0.01; ***P < 0.001; ****P < 0.0001. The data are from three or four independent tests and are presented as the mean ± SD. **F** WT and miR-106b-KO (106b-144) cell lines transfected with EGFP-Parkin were induced with 100 μΜ H_2_O_2_ for 0-18 h and subjected to WB analysis with the indicated antibodies. *N* = 3; *P < 0.05; **P < 0.01; ***P < 0.001; ****P < 0.0001. The data are from three independent tests and are presented as the mean ± SD. **G** Mild and sustained H_2_O_2_ stimulation modulates mitochondrial morphology and triggers mitophagy in a Parkin-dependent manner. Meanwhile, H_2_O_2_ stimulation promotes transcription of the miR-106b-93-25 cluster to regulate mitophagy-associated proteins. H_2_O_2_ reduces GSK3β levels in the nucleus, thus decreasing the phosphorylation of c-Myc at T58, producing an unstable form of c-Myc and resulting in increased c-Myc levels. The miR-106b-93-25 cluster, which is downstream of c-Myc, is increased along with c-Myc, thus inhibiting mitophagy-associated proteins (OPTN, NDP52, MFN2, and MCU) to protect against excessive mitophagy.

Excessive ROS, which is generated by dysfunctional mitochondria, directly damage mitochondrial proteins, lipids, and DNA^38,39^. To verify the protective effect of mitophagy, we prolonged the H_2_O_2_ treatment time to 24 h and then examined cell viability. Cell viability declined after 18–24 h of H_2_O_2_ stimulation without Parkin but was significantly restored in the presence of Parkin (Fig. 6A), indicating that moderate mitophagy has a positive effect on cell survival. Twenty four hours of H_2_O_2_ induction significantly increased LC3BII levels but had no effect on the expression of caspase-3 and cleaved caspase-3 (an apoptosis marker) in both EGFP-Parkin-transfected and EGFP-C1-transfected cells (Fig. 6B, C), suggesting that H_2_O_2_-induced cell death is due to intracellular autophagy rather than caspase-dependent apoptosis (Supplementary Results). Although mitophagy can restrain cell death, the death rates still increased with continued H_2_O_2_ exposure (18 h to 24 h) (Fig. 6A). Cell viability was significantly higher in the EGFP-Parkin cell lines than in the EGFP-Control cell lines after 18–24 h of H_2_O_2_ treatment but still tended to decrease over time (cell viability at 18 h, 85.7%; at 24 h, 46.8%) (Supplementary Fig. 9), suggesting that excessive mitophagy can cause cell death. To further confirm this result, OPTN, the main mitophagy receptor, was deleted in HeLa cells (Supplementary Fig. 10A). After 18 h of H_2_O_2_ stimulation, the levels of TFAM in *OPTN-KO* cells were slightly decreased, while those in WT HeLa cells were dramatically decreased in the presence of Parkin (Fig. 7A), indicating that deletion of OPTN can restrain mitophagy. The cell viability of *OPTN-KO* cells was also apparently higher than that of WT HeLa cells after 18 h of H_2_O_2_ stimulation (Fig. 7B). These results suggest that appropriate inhibition of mitophagy is beneficial for cell survival.

OPTN, NDP52, and MFN2 have been reported to aid in the elimination of impaired mitochondria^2–5^. Excessive mitophagy leads to cell death because of metabolic and bioenergetic collapse^40^, and we have demonstrated that the deletion of OPTN can restrain excessive mitophagy to maintain cell survival. Therefore, we hypothesized that miR-106b and miR-93 can alleviate cell death through the downregulation of OPTN, NDP52, and MFN2. As expected, 24 h after EGFP-Parkin transfection, the viability of both miR-106b- and miR-93-KO cells (106b-32, 93-12) was lower than that of WT cells after 18–24 h of H_2_O_2_ stimulation (Fig. 7C, D). Surprisingly, the viability of miR-25-KO cells (25-106) was also lower than that of WT cells (Fig. 7E). Analysis of data from TargetScanHuman and miRDB websites revealed that no mitophagy-associated proteins are directly targeted by miR-25. Nevertheless, miR-25 can inhibit the mitochondrial calcium uniporter (MCU) by binding to the 3’UTR of MCU mRNA^41^, and inhibition of MCU protects against ischemia/reperfusion injury by inhibiting excessive mitophagy^42^. Consistent with the previous findings^41^, MCU levels were increased in miR-25-KO cells (Supplementary Fig. 10B), illustrating that the reduced cell viability of miR-25-KO cells resulted from upregulation of MCU. As miR-106b plays a predominant role in the regulation of mitophagy-associated proteins, the increased mitophagy in miR-106b-KO cells can further prove that miR-106b negatively regulates mitophagy. As expected, the levels of TFAM and TOMM40 in EGFP-Parkin-transfected WT and miR-106b-KO cells (106b-144) were decreased after 18 h of H_2_O_2_-stimulation; TFAM and TOMM40 levels were, on average, 12% and 24% lower, respectively, in 106b-144 cells than in WT cells (Fig. 7F), indicating that miR-106b has inhibitory effects on mitophagy. Collectively, all of these results reveal that the miR-106b-93-25 cluster protects cells from excessive mitophagy.

## Discussion

Oxidative stress elicited by the accumulation of ROS is an important mechanism of various disease^43–46^. ROS are the major inducers of mitophagy under both pathological and physiological conditions. Increasing evidence has established the causal role of mitophagy in various diseases^47,48^. Hence, elucidating the modulation of ROS-induced mitophagy should provide insights for the treatment of diseases characterized by impaired mitochondria. Our work demonstrates that the signaling molecule H_2_O_2_ induces Parkin-mediated mitophagy and mediates the GSK3β/c-Myc pathway to upregulate the miR-106b-93-25 cluster, which can inhibit the mitophagy receptors OPTN and NDP52 and the Parkin substrate MFN2; thus, our data indicate that ROS-mediated signal transduction and mitophagy regulation correlate.

Here, we also found that OPTN deletion did not change mitochondrial quality but reduced MFN2 expression, leading to mitochondrial fragmentation (Supplementary Results). Unexpectedly, the viabilities of *OPTN-KO* and WT HeLa cells decreased to the same level after 24 h of H_2_O_2_ stimulation (Fig. 7B). It has been reported that overexpression of OPTN can protect against H_2_O_2_-induced cell death and that OPTN knockdown causes neuronal cell death via inappropriate NF-κB activity^49,50^. Therefore, we hypothesized that inappropriate activation of NF-κB might lead to the death of *OPTN-KO* cells 24 h after H_2_O_2_ stimulation.

Interestingly, we extended the application of the CRISPR/Cas9 system to regulate the expression of a miRNA cluster. And we summarized three important conclusions concerning the accurate deletion of a single miRNA in a certain miRNA cluster (Supplementary Results). An article by Lataniotis has demonstrated that the downregulation of miR-25 expression does not affect the miR-106b and miR-93 levels^51^, which was in disagreement with our results. We believe that the different statistical methods led to these deviations. Lataniotis analyzed the overall levels of miR-106b, miR-93, and miR-25 after using the CRISPR/Cas9 system to edit miR-25, however, the knockout efficiency may mask the effects of miR-25 deletion on the other miRNAs. We singled out a few homozygous miR-25-KO cell lines and thus were able to precisely analyze changes in the levels of miR-106b and miR-93.

In summary, we found that mild and sustained H_2_O_2_ stimulation triggers mitophagy in a Parkin-dependent manner; and H_2_O_2_ reduces the accumulation of GSK3β in the nucleus, thus decreasing the phosphorylation of c-Myc at T58 and subsequently increasing c-Myc levels. The levels of the miR-106b-93-25 cluster, which is downstream of c-Myc, were elevated with increasing c-Myc levels, and this cluster inhibits mitophagy-associated proteins (OPTN, NDP52, MFN2, and MCU) to protect against excessive mitophagy (Fig. 7G). Our work highlights a novel mechanism by which mitophagy is triggered and controlled and provides guidance for the editing of clustered miRNAs. Both contributions are of physiological significance for the study of mitochondrial quality control and the development of therapies to treat diseases such as cancer, neurodegeneration, and cardiovascular disease.

## Materials and methods

### Antibodies

The antibodies used for WB and IF included TFAM (WB 1:1 000, IF 1:200, Proteintech, 23996-1-AP), AMBRA1 (WB 1:500, IF 1:100, Abclonal, A1083), MCU (WB 1:1 000, Proteintech, 26312-1-AP), NRF2 (WB 1:1 000, IF 1:200, Proteintech, 16396-1-AP), Beta Tubulin (WB 1:40 000, Proteintech, 66240-1-Ig), CASP3 (WB 1:1 000, Abclonal, A2156), PP2A-Cα/β (WB 1:1 000, IF 1:200, Santa Cruz, SC-80665) GSK3β (WB 1:1 000, IF 1:200, Abclonal, A2081), p-GSK3β-S9 (IF 1:200, Proteintech, 67558-1-Ig), p-c-Myc-S62 (WB 1:1 000, IF 1:200, Abcam, AB185656), AXIN1 (WB 1:1 000, Abclonal, A16019), TOMM20 (WB 1:1 000, IF 1:200, Abclonal, A16896), p-c-Myc-T58 (WB 1:1 000, IF 1:200, Abclonal, AP0080), OPTN (WB 1:2 000, Proteintech, 10837-1-AP) NDP52 (WB 1:1 000, Proteintech, 12229-1-AP), p62/SQSTM1 (WB 1:1 000, Proteintech, 66184-1-Ig), Flag (WB 1:2 000, IF 1:200, Abbkine, A02010), TOMM40 (WB 1:1 000, Proteintech, 18409-1-AP), GFP (WB 1:5 000, Proteintech, 50430-2-AP), LC3B (WB 1:500, Abclonal, A19665), MFN1 (WB 1:1 000, Proteintech, 13798-1-AP), MFN2 (WB 1:1 000, Proteintech, 12186-1-AP), c-Myc (WB 1:1 000, IF 1:200, CST, # 13987), and Lamin B1 (WB 1:1 000, Abbkine, A01090). The secondary antibodies for WB analysis included AffiniPure goat anti-rabbit IgG (H + L) and goat anti-rabbit IgG (H + L) (Promoter Biotechnology, Ltd.). The secondary antibodies for IF included DyLight 488/594/649 AffiniPure goat anti-mouse and goat anti-rabbit antibodies (Abbkine).

### Cell culture and transfection

HeLa and HEK 293T cells were obtained from the China Center for Type Culture Collection (CCTCC, China). Both cell lines were confirmed to be mycoplasma-free and cultured in high-glucose DMEM (HyClone, SH30022.01) supplemented with 1/10 fetal bovine serum (FBS; Tianhang Biotechnology, 11011-8611) and a 1/100 penicillin-streptomycin solution (KeyGen Biotech, KGY0023-100) in CO_2_ incubators (ESCO) at 37 °C in 5% CO_2_. Lipofectamine 2 000 was used for transient transfection and lentiviral transfection, and Lipofectamine RNAiMAX was used for miRNA mimic transfection (Thermo Fisher Scientific, 11668027, 13778030).

### Cloning and plasmids

The PG13-U6-gRNA and Pst1375-NLS-Cas9 plasmids were obtained from Prof. Xiaodong Zhang’s laboratory (College of Life Science, Wuhan University). Then, we optimized PG13-U6-gRNA to make it more efficient. The optimized PG13-U6-gRNA plasmid was named PG13-C-2. The sequences of the oligos used to generate gene-KO constructs targeting the miR-106b-93-25 cluster are listed in Supplementary Table 1. The constructed PG13-C-2-106b, PG13-C-2-93, and PG13-C-2-25 plasmids were separately cotransfected with the Pst1375-NLS-Cas9 plasmid into HeLa cells. After 48 h, the transfected cells were harvested for genome extraction. Then, the amplified products were subjected to sequencing. The presence of overlapping peaks in the target region indicated the effectiveness of vector construction. The transfected cells were diluted and cultured in 96-well plates, and single colonies were collected and examined to obtain cell lines with homozygous deletion.

HeLa cell lines stably expressing EGFP, an EGFP-Parkin fusion protein, and a cox8-flag-TALE-flag fusion protein were established by lentiviral transfection. The lentiviral plasmids PHAGE-Myc-N (RSV), PSPAX2, and PMD2.G were obtained from Prof. Mingzhou Chen’s laboratory (College of Life Science, Wuhan University)^52^. The EGFP sequence, EGFP-Parkin sequence, and cox8-flag-TALE-flag sequence were separately cloned into the PHAGE-Myc-N (RSV) vector, and the resulting constructs were named P-E-2, P-E-P-31, and PHAGE-2175, respectively. shRNA sequences targeting human NRF2 and a control shRNA sequence (listed in Supplementary Table 2) were separately cloned into pLKO.1-puro vectors and the resulting constructs were named pLKO.1-puro-shNRF2-1, pLKO.1-puro-shNRF2-2, and pLKO.1-puro-shCtrl, respectively. Viral packaging and cell screening protocols were performed. Briefly, 5 μg of PSPAX2, 5 μg of PMD2.G, and 10 μg of P-E-2 (P-E-P-31, PHAGE-2175, pLKO.1-puro-shNRF2-2, or pLKO.1-puro-shCtrl) were cotransfected into HEK 293T cells, which were cultured in 10 cm Petri dishes to a confluence of 70%-80%. Forty-eight to 72 h after transfection, cells, and supernatants were collected and centrifuged (4 000 × *g*, 4 °C, 10 min), and the supernatants were filtered through a 0.45 μm membrane filter. The filtered supernatant was then mixed with 1 × 10^6^ HeLa cells in suspension. Puromycin (1 μg/ml) was added to the medium to screen infected cells for 7–10 days. The EGFP-C1 (control), EGFP-Parkin, and mKeima-Red-Mito-7 plasmids were used for transient transfection. The EGFP-C1 plasmid was purchased from Addgene, the mKeima-Red-Mito-7 plasmid was purchased from the MiaoLing Plasmid Sharing Platform, and the EGFP-Parkin plasmid was a gift from Prof. Zhiyin Song (College of Life Science, Wuhan University)^53^.

### Cell treatment

Cells were maintained in a medium containing 100 μM H_2_O_2_ for 12 h, 18 h, or 24 h, and the medium was changed every 6 h to keep the level of H_2_O_2_ constant.

### Mitochondrial staining and morphology analysis

HeLa cells were cultured in confocal Petri dishes (Nest, 801001) and induced with H_2_O_2_ if necessary. After induction, the cells were rinsed with phosphate-buffered saline (PBS) and immediately incubated with DMEM-diluted MitoTracker Red CMXRos (1:10 000, Invitrogen, M7512) for 10 min at 37 °C in the dark. After incubation, the cells were washed with PBS three times for 8 min each time. The mitochondria were imaged by confocal microscopy. The mitochondrial morphology was analyzed with an ImageJ macro tool^54^.

### WB analysis

Cells were rinsed with PBS and then lysed in SDS-PAGE sample loading buffer (2 ×, Beyotime Biotechnology, P0015B). The cell lysate was boiled at 100 °C for 10 min before being subjected to sodium dodecyl sulfate-polyacrylamide gel electrophoresis (SDS-PAGE). The separated proteins were transferred to a PVDF membrane (Merck Millipore, IPVH00010). Then, the PVDF membrane was blocked with a skim milk solution (5% skim milk in 1 × TBST) before being incubated with antibodies and visualized with enhanced chemiluminescence (ECL) substrate (Bio-Rad, 1705062).

### IF analysis

HeLa cells cultured in confocal Petri dishes (Nest, 801001) were rinsed with PBS and immediately fixed with 4% paraformaldehyde (PFA) for 15–20 min at room temperature (RT). The immobilized cells were incubated with Triton X-100 (0.2%) and 2% BSA in sequence to increase cell permeability and block nonspecific antibody-antigen binding, respectively. The cells were incubated with primary antibodies overnight at 4 °C and with secondary antibodies for 2 h at RT. DAPI (5 μg/ml, Beyotime Biotechnology, C1002) was added and allowed to stain cellular nuclei for 10 min at RT. After each incubation, the cells were washed with PBS three times for 8 min each time. The IF-stained cells were detected under confocal microscopy.

### miRNA mimic transfection

miRNA mimics (a miR-106b mimic and a miR-93 mimic) and a mimic NC were designed by RiboBio Biotechnology. Each construct (200 nM) was separately transfected into cells cultured in 12-well plates with 1.5 μl of Lipofectamine RNAiMAX. Forty-eight hours after transfection, cells were harvested for WB analysis.

### miRNA northern blot analysis

miRNAs were extracted according to the instructions of a miRNA Purification Kit (CWBIO, CW0627S). DIG-labeled locked nucleic acid (LNA) probes were used to detect the miRNAs and U6 (as a control)^55^. Based on a DIG-High Prime DNA Labeling and Detection Starter Kit II (Roche 11585614910), the experimental conditions were optimized for convenient and efficient miRNA detection. The LNA-labeled primers used in our study are listed in Supplementary Table 3. Urea-denaturing gels (15%) were prepared by mixing 5 ml of acrylamide/bis-acrylamide, 2 ml of 5 × TBE, 3 ml of urea (0.083 g/ml), 40 μl of APS (10%) and 4 μl of TEMED. The gels were prerun for 30 min at 200 V in 1 × TBE in a vertical Mini-PROTEAN tank (Bio-Rad). Total RNA (5-20 μg) was mixed with RNA Loading Dye (2 ×, NEB, B0363S), denatured for 5 min at 95 °C, and then immediately placed on ice. The RNA samples were run at 200 V until the bromophenol blue dye front reached the bottom of the gel. After electrophoresis, the RNA samples were transferred to nylon membranes (Roche, 11209272001) at 10–15 V (1 h, 0.5 × TBE) in a Mini Trans-Blot module (Bio-Rad). After the transfer was complete, the membrane was gently wrapped with dry filter paper for drying. Then, the membrane was exposed to UV (120 mJ/cm^2^, 1 min) for RNA cross-linking. Membrane prehybridization was performed in DIG Easy Hyb buffer (Roche) at 50 °C for 30 min, after which the membrane was removed from the buffer. The LNA-labeled probes were denatured at 95 °C for 1 min. The denatured LNA-labeled probes were added to DIG Easy Hyb buffer (10 pmol/μl 106b-dig-LNA, 93-dig-LNA, or 25-dig-LNA in DIG Easy Hyb buffer; 2 pmol/μl U6-dig-LNA in DIG Easy Hyb buffer) to prepare the hybridization buffer. After prehybridization, the membrane was incubated with the hybridization buffer containing the LNA-labeled probes (42 °C, ON). The hybridization buffer (which could be reused) was removed the next day, and the membrane was washed twice in 2 × Stringency Wash Solution (2 × SSC with 0.1% SDS) for 15 min at 50 °C, twice in 0.5 × Stringency Wash Solution (0.5 × SSC and 0.1% SDS) for 10 min at 50 °C, and finally, washed once in 0.1 × Stringency Wash Solution (0.1 × SSC and 0.1% SDS) for 5 min at 50 °C. Images of U6 and the miRNAs were captured with the Roche DIG-High Primer DNA Labeling and Detection Starter Kit II following the manufacturer’s protocol with a ChemiDoc XRS + (Bio-Rad).

### RNA extraction and RT-qPCR

Total RNA was extracted using TRIzol® Reagent (Invitrogen, 15596018) following the manufacturer’s instructions. The reverse transcription (RT) reaction was performed according to the directions of a FastQuant RT Kit (with gDNase) (Tiangen, KR106-02). The stem-loop primers (synthesized by GenScript, Inc.) used in the RT reaction for miRNA detection are listed in Supplementary Table 4. The RT-qPCR system was prepared following the instructions of AceQ® qPCR SYBR® Green Master Mix (Vazyme, Q111-02). The ΔCT value was measured and recorded using an ABI 7500 instrument. The relative quantity of each miRNA was normalized to the quantity of U6 using the ΔΔCT method. Similarly, the relative quantity of each mRNA was normalized to the quantity of Tubulin using the ΔΔCT method. The RT-qPCR primers used in our study are listed in Supplementary Table 5.

### Double-luciferase reporter assay

The 3’UTRs of the human *OPTN*, *MFN2,* and *CALCOCO2* genes were amplified by three pairs of primers, which are listed in Supplementary Table 6. Then, these 3’UTRs were cloned into the psiCHECK2.0 plasmid (purchased from Addgene). Site-directed mutagenesis was performed in the psiCHECK2-h*OPTN*-3’UTR, psiCHECK2-h*MFN2*-3’UTR, and psiCHECK2-h*NDP52*-3’UTR plasmids; specifically, the miR-106b and miR-93 target sites were disrupted. HeLa cells were cotransfected with 400 ng of reporter plasmid (or mutant reporter plasmid) and 50 pmol of miR-106b (or miR-93 mimic) using Lipofectamine 2 000 in 24-well plates. Forty-eight hours after transfection, the firefly and Renilla luciferase activity levels were measured using a *TransDetect* Double-Luciferase Reporter Assay Kit (TransGen, FR201-01) according to the manufacturer’s instructions. The results are shown as the fluorescence intensity of Renilla luciferase normalized to that of firefly luciferase.

### Cell viability detection

Twelve hours after transfection, 2–3 × 10^4^ cells were seeded in 96-well plates. The cells were then treated with 100 μM H_2_O_2_ for different periods of time. Wells containing culture medium without cells were used as negative controls. The samples were prepared following the directions of a CellTiter-Lumi™ Luminescent Cell Viability Assay Kit (Beyotime Biotechnology, C0065S), and cell viability was evaluated with a multimode microplate detection system (Molecular Devices, SpectraMax® i3x).

### Measurement of intracellular ROS

Intracellular ROS were detected with a ROS Assay Kit (Beyotime Biotechnology, S0033) in accordance with the manufacturer’s instructions. The cells were transfected with mCherry-C1 (as a control) and mCherry-Parkin plasmids. After 24 h, the cells were treated with 100 μΜ H_2_O_2_ for 0 h, 4 h, 8 h, or 12 h and then incubated with DCFH-DA (an oxidation-sensitive fluorescent probe, 10 μM) at 37 °C for 20 min in the dark. The cells were collected, and the fluorescence intensity was measured with a flow cytometer (BD FACSCelesta). FlowJo software was used for data analysis.

### Nuclear and cytoplasmic protein isolation

An optimized protocol based on the instructions of a Nuclear and Cytoplasmic Protein Extraction Kit (Beyotime Biotechnology, P0027) was used to separate the nuclear portion from the cytoplasmic portion. Cells were digested with a 0.02% EDTA solution at 37 °C for 2 min and centrifuged at 4 °C for 5 min. The precipitate was lysed by cytosolic protein extraction reagent A containing 1% PMSF for 10–15 min on ice. Cytosolic protein extraction reagent B was then added, and the suspension was kept on ice for another 5 min. After centrifugation, the supernatant (containing cytoplasmic proteins) was transferred to a precooled tube. The pellet was washed once with a mixed solution of cytosolic protein extraction reagents A and B (20:1), and SDS-PAGE sample loading buffer containing SDS was added to lyse the nuclei.

### Statistical analysis

Unless otherwise stated, data were from at least three independent tests and presented as the means ± standard deviation (SD). Data were statistically analyzed using Prism (Version 7; GraphPad) software. Two-group comparisons were performed by Student’s *t*-test with Welch’s correction and did not assume equal SDs. All data were evaluated with two-tailed tests. A *p*-value <0.05 was assessed as statistical significance.

## Supplementary information

Supplementary Results

Supplementary Figure Legends

Supplementary Figure 1

Supplementary Figure 2

Supplementary Figure 3

Supplementary Figure 4

Supplementary Figure 5

Supplementary Figure 6

Supplementary Figure 7

Supplementary Figure 8

Supplementary Figure 9

Supplementary Figure 10

Supplementary Tables

## References

[1] Lemasters JJ (2005). Selective mitochondrial autophagy, or mitophagy, as a targeted defense against oxidative stress, mitochondrial dysfunction, and aging. Rejuvenat. Res..

[2] Chen Y, Dorn GW (2013). PINK1-phosphorylated mitofusin 2 is a Parkin receptor for culling damaged mitochondria. Science.

[3] Wong YC, Holzbaur EL (2014). Optineurin is an autophagy receptor for damaged mitochondria in parkin-mediated mitophagy that is disrupted by an ALS-linked mutation. Proc. Natl Acad. Sci. USA.

[4] Heo JM, Ordureau A, Paulo JA, Rinehart J, Harper JW (2015). The PINK1-PARKIN Mitochondrial Ubiquitylation Pathway Drives a Program of OPTN/NDP52 Recruitment and TBK1 Activation to Promote Mitophagy. Mol. Cell.

[5] Lazarou M (2015). The ubiquitin kinase PINK1 recruits autophagy receptors to induce mitophagy. Nature.

[6] Itoh K, Nakamura K, Iijima M, Sesaki H (2013). Mitochondrial dynamics in neurodegeneration. Trends Cell Biol..

[7] Knott AB, Perkins G, Schwarzenbacher R, Bossy-Wetzel E (2008). Mitochondrial fragmentation in neurodegeneration. Nat. Rev. Neurosci..

[8] Deng Z (2017). Autophagy receptors and neurodegenerative diseases. Trends Cell Biol..

[9] Allen GF, Toth R, James J, Ganley IG (2013). Loss of iron triggers PINK1/Parkin-independent mitophagy. EMBO Rep..

[10] Wang Y, Nartiss Y, Steipe B, McQuibban GA, Kim PK (2012). ROS-induced mitochondrial depolarization initiates PARK2/PARKIN-dependent mitochondrial degradation by autophagy. Autophagy.

[11] Lin MT, Beal MF (2006). Mitochondrial dysfunction and oxidative stress in neurodegenerative diseases. Nature.

[12] Stone JR, Yang S (2006). Hydrogen peroxide: a signaling messenger. Antioxid. Redox Signal..

[13] Saccani A (2000). Redox regulation of chemokine receptor expression. Proc. Natl Acad. Sci. USA.

[14] Bartel DP (2009). MicroRNAs: target recognition and regulatory functions. Cell.

[15] Li W (2014). MicroRNA-137 Is a Novel Hypoxia-responsive MicroRNA That Inhibits Mitophagy via Regulation of Two Mitophagy Receptors FUNDC1 and NIX. J. Biol. Chem..

[16] Kim J (2016). miR-27a and miR-27b regulate autophagic clearance of damaged mitochondria by targeting PTEN-induced putative kinase 1 (PINK1). Mol. Neurodegener..

[17] Frank M (2012). Mitophagy is triggered by mild oxidative stress in a mitochondrial fission dependent manner. Biochim. Biophys. Acta.

[18] Giorgio M, Trinei M, Migliaccio E, Pelicci PG (2007). Hydrogen peroxide: a metabolic by-product or a common mediator of ageing signals?. Nat. Rev. Mol. Cell Biol..

[19] Zhang H (2009). Oxidative stress induces parallel autophagy and mitochondria dysfunction in human glioma U251 cells. Toxicol. Sci..

[20] Iqbal S, Hood DA (2014). Oxidative stress-induced mitochondrial fragmentation and movement in skeletal muscle myoblasts. Am. J. Physiol. Cell Physiol..

[21] Narendra D, Tanaka A, Suen DF, Youle RJ (2009). Parkin-induced mitophagy in the pathogenesis of Parkinson disease. Autophagy.

[22] Heo SR, Han AM, Kwon YK, Joung I (2009). p62 protects SH-SY5Y neuroblastoma cells against H_2_O_2_-induced injury through the PDK1/Akt pathway. Neurosci. Lett..

[23] Jain A (2010). p62/SQSTM1 is a target gene for transcription factor NRF2 and creates a positive feedback loop by inducing antioxidant response element-driven gene transcription. J. Biol. Chem..

[24] McLelland GL, Fon EA (2018). MFN2 retrotranslocation boosts mitophagy by uncoupling mitochondria from the ER. Autophagy.

[25] Motohashi H, Yamamoto M (2004). Nrf2-Keap1 defines a physiologically important stress response mechanism. Trends Mol. Med..

[26] Jo C (2014). Nrf2 reduces levels of phosphorylated tau protein by inducing autophagy adaptor protein NDP52. Nat. Commun..

[27] O’Donnell KA, Wentzel EA, Zeller KI, Dang CV, Mendell JT (2005). c-Myc-regulated microRNAs modulate E2F1 expression. Nature.

[28] Zhao ZN (2012). TSA suppresses miR-106b-93-25 cluster expression through downregulation of MYC and inhibits proliferation and induces apoptosis in human EMC. PLoS ONE.

[29] Sun Y (2009). Expression profile of microRNAs in c-Myc induced mouse mammary tumors. Breast Cancer Res. Treat..

[30] Petrocca F, Vecchione A, Croce CM (2008). Emerging role of miR-106b-25/miR-17-92 clusters in the control of transforming growth factor beta signaling. Cancer Res..

[31] Poliseno L (2010). Identification of the miR-106b~25 microRNA cluster as a proto-oncogenic PTEN-targeting intron that cooperates with its host gene MCM7 in transformation. Sci. Signal..

[32] Yap CS, Peterson AL, Castellani G, Sedivy JM, Neretti N (2011). Kinetic profiling of the c-Myc transcriptome and bioinformatic analysis of repressed gene promoters. Cell Cycle.

[33] Yeh E (2004). A signalling pathway controlling c-Myc degradation that impacts oncogenic transformation of human cells. Nat. Cell Biol..

[34] Sears RC (2004). The life cycle of C-myc: from synthesis to degradation. Cell Cycle.

[35] Arnold HK, Sears RC (2008). A tumor suppressor role for PP2A-B56α through negative regulation of c-Myc and other key oncoproteins. Cancer Metastasis Rev..

[36] Cianfanelli V (2015). AMBRA1 links autophagy to cell proliferation and tumorigenesis by promoting c-Myc dephosphorylation and degradation. Nat. Cell Biol..

[37] Sears R (2000). Multiple Ras-dependent phosphorylation pathways regulate Myc protein stability. Genes Dev..

[38] Youle RJ, van der Bliek AM (2012). Mitochondrial fission, fusion, and stress. Science.

[39] Marin-Garcia J, Akhmedov AT (2016). Mitochondrial dynamics and cell death in heart failure. Heart Fail Rev..

[40] Kim EH, Choi KS (2014). A critical role of superoxide anion in selenite-induced mitophagic cell death. Autophagy.

[41] Marchi S (2013). Downregulation of the mitochondrial calcium uniporter by cancer-related miR-25. Curr. Biol..

[42] Yu S (2016). Inhibition of mitochondrial calcium uniporter protects neurocytes from ischemia/reperfusion injury via the inhibition of excessive mitophagy. Neurosci. Lett..

[43] Liu Z, Zhou T, Ziegler AC, Dimitrion P, Zuo L (2017). Oxidative stress in neurodegenerative diseases: from molecular mechanisms to clinical applications. Oxid. Med. Cell Longev..

[44] Davalli P, Mitic T, Caporali A, Lauriola A, D’Arca D (2016). ROS, cell senescence, and novel molecular mechanisms in aging and age-related diseases. Oxid. Med. Cell Longev..

[45] Prasad S, Gupta SC, Tyagi AK (2017). Reactive oxygen species (ROS) and cancer: role of antioxidative nutraceuticals. Cancer Lett..

[46] Ochoa CD, Wu RF, Terada LS (2018). ROS signaling and ER stress in cardiovascular disease. Mol. Asp. Med..

[47] Palikaras K, Tavernarakis N (2012). Mitophagy in neurodegeneration and aging. Front Genet.

[48] Rodolfo C, Campello S, Cecconi F (2018). Mitophagy in neurodegenerative diseases. Neurochem. Int..

[49] De Marco N, Buono M, Troise F, Diez-Roux G (2006). Optineurin increases cell survival and translocates to the nucleus in a Rab8-dependent manner upon an apoptotic stimulus. J. Biol. Chem..

[50] Akizuki M (2013). Optineurin suppression causes neuronal cell death via NF-kappaB pathway. J. Neurochem..

[51] Lataniotis L (2017). CRISPR/Cas9 editing reveals novel mechanisms of clustered microRNA regulation and function. Sci. Rep..

[52] Hu Z (2018). Inclusion bodies of human parainfluenza virus type 3 inhibit antiviral stress granule formation by shielding viral RNAs. PLoS Pathog..

[53] Jian F (2018). Sam50 regulates PINK1-Parkin-mediated mitophagy by controlling PINK1 stability and mitochondrial morphology. Cell Rep..

[54] Valente AJ, Maddalena LA, Robb EL, Moradi F, Stuart JA (2017). A simple ImageJ macro tool for analyzing mitochondrial network morphology in mammalian cell culture. Acta Histochem..

[55] Kim SW (2010). A sensitive non-radioactive northern blot method to detect small RNAs. Nucleic Acids Res..

