# Supplementary material for: Oxidative stress-induced mitophagy is suppressed by the miR-106b-93-25 cluster in a protective manner
Source: Cell Death Dis. 2021 Feb 24;12(2):209. doi: 10.1038/s41419-021-03484-3 (PMC7904769; doi:10.1038/s41419-021-03484-3)
Supplement: Supplementary file 1 — Supplementary Results [file 41419_2021_3484_MOESM1_ESM.docx]

**Oxidative stress-induced mitophagy is suppressed by the miR-106b-93-25 cluster in a protective manner**

Cheng Zhang^1,2,3^, Pengqing Nie^1,2,4^, Chunliu Zhou^1^, Yue Hu^1^, Suling Duan^1^, Meijia Gu^1^, Dongxu Jiang^1^, Yunfu Wang^2^, Zixin Deng^1^, Jincao Chen^3^, Shi Chen^1,2,3^, Lianrong Wang^1,2,3*^

^1^*Key Laboratory of Combinatorial Biosynthesis and Drug Discovery, Ministry of Education, School of Pharmaceutical Sciences, Wuhan University, Wuhan 430071, Hubei, China*

^2^*Taihe Hospital, Hubei University of Medicine, Shiyan 442000, Hubei, China*

^3^*Brain Center, Department of Neurosurgery, Zhongnan Hospital, Wuhan 430071, China*

^4^*Department of Burn and Plastic Surgery, Division of Wound Repair, Shenzhen Institute of Translational Medicine,* *the First Affiliated Hospital, Shenzhen University, Shenzhen, 518035, Guangdong, China*

*For correspondence: Lianrong Wang, Chu-Tian Distinguished Professor, lianrong@whu.edu.cn

**Supplementary Results**

**H_2_O_2_ induction modulates mitochondrial morphology in the CFTF cell line.**

To further confirm the changes in mitochondrial morphology, EGFP-Parkin and EGFP-C1 were transfected into the CFTF cell line (stably expressing a **c**ox8-**f**lag-**T**ALE-**f**lag fusion protein), which can bind to mitochondrial DNA via transcription activator-like effectors (TALEs) (Supplementary Fig. 1B, C); CFTF cells were then stimulated with 100 μM H_2_O_2_ for 0-12 h. As expected, the mean branch length and mean network size decreased while the number of individual mitochondria increased in EGFP-C1-transfected cells. By contrast, in EGFP-Parkin-transfected cells, the number of individual and network mitochondria decreased and the mean network size was reduced (Supplementary Fig. 2A, B), suggesting that mild and sustained H_2_O_2_ stimulation can induce a change in mitochondrial morphology.

**Analysis of discordant or concordant changes in mRNA and protein abundance of OPTN, NDP52 and MFN2.**

H_2_O_2_ treatment downregulated OPTN and MFN2 in both EGFP-Parkin-transfected and EGFP-C1-transfected cells, suggesting that the decreases in OPTN and MFN2 levels were not caused by Parkin-mediated mitophagy. The lack of changes in MFN1 levels suggested that the decreases in MFN2 levels were not caused by mitochondrial morphology changes. In addition, NDP52 was downregulated in EGFP-Parkin-transfected cells after 18 h of H_2_O_2_ induction. Furthermore, we measured the mRNA levels of these genes by real-time fluorescent quantitative PCR (RT-qPCR) and found that mRNA levels of NDP52 and p62 were almost unchanged after 12 h of H_2_O_2_ treatment in both EGFP-Parkin-transfected and EGFP-C1-transfected cells; OPTN mRNA levels were slightly decreased after H_2_O_2_ treatment only in EGFP-C1-transfected cells (Supplementary Fig. 5A), indicating that H_2_O_2_ induction had little effect on the transcription of these genes. Combined with a decrease in the protein levels of OPTN and NDP52, we speculate that the translation of OPTN and NDP52 is inhibited. The mRNA levels of MFN2 and MFN1 showed almost identical declines after 12 h of H_2_O_2_ treatment (Supplementary Fig. 5A), indicating that their transcription was inhibited. Considering that the protein levels of MFN2 were significantly lower than that of MFN1 upon H_2_O_2_-induction, we speculate that not only the transcription of MFN2 is inhibited, but also the translation is inhibited.

**H_2_O_2_-induced cell death is due to intracellular autophagy rather than caspase-dependent apoptosis.**

Autophagy and apoptosis can lead to cell death in distinct ways[^1^](#_ENREF_1). To investigate what caused the cell death, an autophagy marker LC3B was assessed by WB in H_2_O_2_-induced cells. LC3BII exhibited dramatic upregulation and the LC3BII/LC3BI ratio was obviously increased after 24 h of H_2_O_2_ treatment, suggesting that levels of autophagy were increased in both EGFP-Parkin-transfected and EGFP-C1-transfected cells (Fig. 6b). More importantly, LC3BII levels and the LC3BII/LC3BI ratio were more obviously increased in EGFP-C1-transfected control cells than in EGFP-Parkin-transfected cells (Fig. 6b); this indicated that, compared to EGFP-Parkin-transfected cells, the lower cell viability of EGFP-C1-transfected control cells under oxidative stress was due to more intense autophagy in these cells, and that appropriate mitophagy has a protective effect on cells. Moreover, there was little change in caspase-3 levels and cleaved caspase-3 (an apoptosis marker) levels remained low, with minimal change after 18-24 h of H_2_O_2_-treatment in both EGFP-Parkin-transfected cells and EGFP-C1-transfected control cells (Fig. 6c). These results suggest that H_2_O_2_-induced cell death is due to intracellular autophagy rather than caspase-dependent apoptosis.

**OPTN deletion alters mitochondrial morphology but does not alter mitochondrial quality.**

To date, five different autophagy receptors (NBR1, NDP52, OPTN, p62/SQSTM1 and TAX1BP1) have been shown to translocate to damaged mitochondria during PINK1/Parkin mitophagy[^2^](#_ENREF_2)^,^[^3^](#_ENREF_3). Among these five autophagy receptors, OPTN acts as the primary mitophagy receptor[^4^](#_ENREF_4). Moreover, the lack of change in the expression of TOMM40 in *OPTN-KO* HeLa cells illustrated that OPTN deletion had no effect on mitochondrial quality (Supplementary Fig. 10A, D). One Parkin substrate is MFN2, which has been proven to be crucial for PINK1/Parkin-triggered mitophagy. After being phosphorylated by PINK1, MFN2 promotes Parkin recruitment to damaged mitochondria for MFN2 ubiquitination^[5](#_ENREF_5)^. Mitochondrial dissociation from the endoplasmic reticulum (ER), which requires MFN2 ubiquitination, gives Parkin access to VDAC1 (another Parkin substrate) for its ubiquitination[^6^](#_ENREF_6). In the current study, MFN2 was upregulated while NDP52 was downregulated in *OPTN-KO* HeLa cells (Supplementary Fig. 10C, D). Furthermore, mitochondrial fragmentation occurred in *OPTN-KO* HeLa cells regardless of Parkin expression (Supplementary Fig. 10E, F), showing that OPTN deletion can cause mitochondrial fragmentation. It has been reported that increases in MFN2 levels damage ER-mitochondrial contacts immediately after inducing mitochondrial fragmentation via activation of Drp1 (ref. [7](#_ENREF_7)). Our results reveal that OPTN deletion elevates the levels of MFN2, which induces mitochondrial fragmentation. However, how OPTN deletion regulates MFN2 requires further study.

**Accurate deletion of each specific miRNA in the miR-106b-93-25 cluster by CRISPR/Cas9 gene editing technology.**

Initial reports demonstrated that CRISPR/Cas9 is a simple and efficient tool for silencing miRNA genes[^8^](#_ENREF_8)^,^[^9^](#_ENREF_9). Further studies have applied the CRISPR/Cas9 system to plant miRNA silencing and deletion of the largest miRNA cluster in mice[^10-12^](#_ENREF_10). Recently, CRISPR/Cas9 was used to investigate clustered miRNA regulation[^13^](#_ENREF_13). We knocked out each individual miRNA in the miR-106b-93-25 cluster. We showed by northern blot miRNA analysis that the three miRNAs were successfully knocked out, and that the other two miRNAs were still expressed in the cells with KO of one miRNA (Supplementary Fig. 6E-G). To determine whether the levels of the other two miRNAs changed when each miRNA was knocked out individually, we used RT-qPCR to measure the expression of the other miRNAs. All results are shown in **Supplementary Fig. 6H-J**. The levels of miR-93, which is adjacent to miR-106b, were decreased to different extents in the miR-106b-KO cell lines, while the spacing miR-25 was changed irregularly. However, when the middle miRNA, miR-93, was knocked out, the levels of both of the adjacent miRNAs, miR-106b and miR-25, declined to varying degrees. As expected, the miR-25-KO cells had decreased miR-93 levels and irregular changes in miR-106b, with the exception of one of the cell lines, 25-186. In this cell line, the adjacent miRNA, miR-93, changed slightly; only 5 bases in the DNA sequence of the miR-25 gene were deleted, far fewer than the numbers deleted in the other cell lines (Supplementary Fig. 6D). Taken together, these findings extended the application of CRISPR/Cas9 methods to the accurate deletion of a single miRNA in a certain miRNA cluster. The following three important conclusions emerged: (1) each miRNA in a miRNA cluster can be entirely knocked out by CRISPR/Cas9; (2) knockout of a fringe miRNA in the miRNA cluster can reduce the levels of the adjacent miRNAs depending on the number of bases deleted, but the influence on spacing miRNAs is random (causing upregulation, downregulation or no change); and (3) knockout of the middle miRNA in the miRNA cluster can reduce the levels of adjacent miRNAs. Furthermore, we speculate that a larger distance between two miRNAs is associated with a smaller impact on one miRNA when the other miRNA is knocked out.

**Supplementary References**

1. Clarke PG. Developmental cell death: morphological diversity and multiple mechanisms. *Anat Embryol (Berl)* **181**, 195-213 (1990).

2. Sarraf SA, Raman M, Guarani-Pereira V, Sowa ME, Huttlin EL, Gygi SP et al. Landscape of the PARKIN-dependent ubiquitylome in response to mitochondrial depolarization. *Nature* **496**, 372-376 (2013).

3. Chan NC, Salazar AM, Pham AH, Sweredoski MJ, Kolawa NJ, Graham RL et al. Broad activation of the ubiquitin-proteasome system by Parkin is critical for mitophagy. *Hum Mol Genet* **20**, 1726-1737 (2011).

4. Heo JM, Ordureau A, Paulo JA, Rinehart J & Harper JW. The PINK1-PARKIN Mitochondrial Ubiquitylation Pathway Drives a Program of OPTN/NDP52 Recruitment and TBK1 Activation to Promote Mitophagy. *Mol Cell* **60**, 7-20 (2015).

5. Chen Y & Dorn GW. PINK1-Phosphorylated Mitofusin 2 Is a Parkin Receptor for Culling Damaged Mitochondria. *Science* **340**, 471-475 (2013).

6. McLelland GL, Goiran T, Yi W, Dorval G, Chen CX, Lauinger ND et al. Mfn2 ubiquitination by PINK1/parkin gates the p97-dependent release of ER from mitochondria to drive mitophagy. *Elife* **7** (2018).

7. Puri R, Cheng XT, Lin MY, Huang N & Sheng ZH. Mul1 restrains Parkin-mediated mitophagy in mature neurons by maintaining ER-mitochondrial contacts. *Nat Commun* **10**, 3645 (2019).

8. Zhao Y, Dai Z, Liang Y, Yin M, Ma K, He M et al. Sequence-specific inhibition of microRNA via CRISPR/CRISPRi system. *Sci Rep* **4**, 3943 (2014).

9. Chang H, Yi B, Ma R, Zhang X, Zhao H & Xi Y. CRISPR/cas9, a novel genomic tool to knock down microRNA in vitro and in vivo. *Sci Rep* **6**, 22312 (2016).

10. Jacobs TB, LaFayette PR, Schmitz RJ & Parrott WA. Targeted genome modifications in soybean with CRISPR/Cas9. *BMC Biotechnol* **15**, 16 (2015).

11. Zhou J, Deng K, Cheng Y, Zhong Z, Tian L, Tang X et al. CRISPR-Cas9 Based Genome Editing Reveals New Insights into MicroRNA Function and Regulation in Rice. *Front Plant Sci* **8**, 1598 (2017).

12. Han J, Zhang J, Chen L, Shen B, Zhou J, Hu B et al. Efficient in vivo deletion of a large imprinted lncRNA by CRISPR/Cas9. *RNA Biol* **11**, 829-835 (2014).

13. Lataniotis L, Albrecht A, Kok FO, Monfries CAL, Benedetti L, Lawson ND et al. CRISPR/Cas9 editing reveals novel mechanisms of clustered microRNA regulation and function. *Sci Rep* **7**, 8585 (2017).
