# Supplementary material for: Oxidative stress-induced mitophagy is suppressed by the miR-106b-93-25 cluster in a protective manner
Source: Cell Death Dis. 2021 Feb 24;12(2):209. doi: 10.1038/s41419-021-03484-3 (PMC7904769; doi:10.1038/s41419-021-03484-3)
Supplement: Supplementary file 2 — Supplementary Figure Legends [file 41419_2021_3484_MOESM2_ESM.docx]

**Supplementary Figure Legends**

**Supplementary Figure 1. Construction of transiently transfected and stably expressing cell lines.**

**(A)** Cells were transfected with EGFP-C1 or EGFP-Parkin plasmids. After 24 h, the cells were treated with 100 μΜ H_2_O_2_ for 0 h, 12 h or 18 h. The samples were immunoblotted with an anti-EGFP antibody. Tubulin was used as an endogenous control.

**(B)** The schematic shows the cox8-flag-TALE-flag fusion protein bound to mitochondrial DNA by TALEs (which recognize DNA sequences through a tandem-repeat domain). The binding site is located in the *MTND6* gene locus.

**(C)** A CFTF cell line (stably expressing a cox8-flag-TALE-flag fusion protein) was immunostained with an anti-flag antibody (red). Scale bar, 10 μm.

**(D)** Immunoblot analysis of two stable cell lines (EGFP-Control and EGFP-Parkin) with an EGFP antibody.

**(E)** Lysates from shCtrl, shNRF2-1 and shNRF2-2 cells were used for NRF2 immunoblotting. The protein levels were analyzed with the indicated antibodies; Tubulin was used as an endogenous control.

**Supplementary Figure 2. Mild and sustained H_2_O_2_ stimulation induces changes in mitochondrial morphology.**

**(A)** The CFTF cell line (Supplementary Figure 1B and C) was transfected with EGFP-C1 or EGFP-Parkin. After 24 h, the cells were induced with 100 μΜ H_2_O_2_ for 12 h and immunofluorescently stained with an anti-flag antibody (red). Scale bar, 10 μm.

**(B)** Mitochondrial morphology was quantitatively analyzed using the MiNA ImageJ macro tool based on fluorescent images of flag (~20 cells for each analysis, Supplementary Figure 2A). Unpaired *t-*test; ns, not significant; *P < 0.05; **P < 0.01; ***P < 0.001; ****P < 0.0001. The data are presented as the mean ± SD.

**Supplementary Figure 3. Mild and sustained stimulation with H_2_O_2_ induces Parkin-mediated mitophagy.**

**(A)** After transfection with EGFP-C1 or EGFP-Parkin, cells were incubated with 100 μΜ H_2_O_2_ for 0 h, 12 h or 18 h. The cells were immunostained with an anti-TFAM antibody (red). The fluorescence intensity of TFAM was analyzed with ImageJ. Scale bar, 10 μm. N = 3; ns, not significant; ****P <0.0001. Data from three independent tests were collected for statistical analysis (mean ± SD).

**(B)** Two stable cell lines (EGFP-Control and EGFP-Parkin) were stimulated with 100 μΜ H_2_O_2_ for 0 h, 12 h or 18 h. TFAM and TOMM40 levels were evaluated by WB analysis, with Tubulin used as an endogenous control. N = 3; ns, not significant; *P < 0.05; ***P <0.001. Data from three independent tests were collected for statistical analysis (mean ± SD).

**Supplementary Figure 4. Mild and sustained stimulation with H_2_O_2_ modulates mitophagy-associated protein levels.**

EGFP-Control and EGFP-Parkin cell lines were treated with 100 μΜ H_2_O_2_ for 0 h, 12 h or 18 h. The levels of the mitophagy-associated proteins MFN2, OPTN and NDP52 were evaluated by WB analysis. Tubulin was used as an endogenous control. N = 3; ns, not significant; **P < 0.01; ***P <0.001. Data from three independent tests were collected for statistical analysis (mean ± SD).

**Supplementary Figure 5. The levels of human gene mRNA (*OPTN, MFN2, NDP52/CALCOCO2, p62/SQSTM1* and *MFN1*) and miRNAs (miR-106b, miR-93 and miR-25) were measured by RT-qPCR.**

**(A)** RT-qPCR analysis of the mRNA levels of the indicated genes (*OPTN, MFN2, NDP52/CALCOCO2, p62/SQSTM1* and *MFN1*) in EGFP-C1- and EGFP-Parkin-transfected cells incubated in 100 μM H_2_O_2_ medium for 0 or 12 h. The data were normalized to human *Tubulin* gene expression and analyzed with the 2^-ΔΔCT^ method. N = 3; ns, not significant;*P < 0.05; **P < 0.01; ***P <0.001. Data from three independent tests were collected for statistical analysis (mean ± SD).

**(B)** Cells were transfected with plasmids encoding EGFP (as a control) or EGFP-Parkin. miRNA RT-qPCR was used to detect the expression of miR-106b, miR-93 and miR-25 after H_2_O_2_ induction (100 μΜ; 0 h or 12 h). U6 acted as a reference gene, and the data were analyzed with the 2^-ΔΔCT^ method. N = 3; *P < 0.05; **P < 0.01; ***P <0.001. Data from three independent tests were collected for statistical analysis (mean ± SD).

**Supplementary Figure 6. CRISPR/Cas9 gene editing technology was used to knock out miR-106b, miR-93 and miR-25.**

**(A)** Schematic of the miR-106b-93-25 cluster. The underlined sequences bind to each gRNA for miRNA deletion. The protospacer adjacent motif (PAM) sequences are indicated by oblique and bolded letters.

**(B)** Alignment of WT and miR-106b-KO cell line sequences using DNAMAN software.

**(C)** Alignment of WT and miR-93-KO cell line sequences.

**(D)** Alignment of WT and miR-25-KO cell line sequences.

**(E, F, G)** miRNAs were extracted for miRNA northern blot analysis with LNA-labeled primers (106b-dig-LNA, 93-dig-LNA, 25-dig-LNA and U6-dig-LNA (control)). The levels of mature miR-93 and miR-25 were detected in each miR-106b-deleted cell line (E); the levels of mature miR-106b and miR-25 were detected in each miR-93-deleted cell line (F); and the levels of mature miR-106b and miR-93 were detected in each miR-25-deleted cell line (G).

**(H, I, J)** RT-qPCR was used to measure the expression of miR-106b, miR-93 and miR-25 in WT and miR-106b/93/25-KO cell lines. U6 was used as a reference gene. N = 3. The data were analyzed with the 2^-ΔΔCT^ method; data from three independent tests were collected for statistical analysis.

**Supplementary Figure 7. H_2_O_2_ stimulation promotes an increase in intracellular ROS concentration.**

**(A)** Cells were transfected with mCherry-C1 (as a control) or mCherry-Parkin plasmids. Twenty-four hours later, the cells were treated with 100 μΜ H_2_O_2_ for 0 h, 4 h, 8 h or 12 h. Intracellular ROS levels were detected with DCFH-DA (a redox-sensitive dye) and measured by FACS.

**(B)** Cells transfected with EGFP-C1 or EGFP-Parkin were incubated with 100 μΜ H_2_O_2_ for 0 h or 12 h. The cells were immunostained with an anti-NRF2 antibody (red). Scale bars, 10 μm.

**Supplementary Figure 8. PP2A levels are unchanged and endonuclear p-GSK3β-S9 aggregates in the c-Myc degradation pathway.**

**(A)** Cells were transfected with EGFP-C1 or EGFP-Parkin and stimulated with 100 μΜ H_2_O_2_ for 0 h, 6 h or 12 h. Then, nuclear and cytoplasmic proteins were isolated for WB analysis. The protein levels were analyzed with PP2A antibodies. N = 3; ns, not significant. The data are from three independent tests and are presented as the mean ± SD.

**(B)** HeLa cells were transfected with EGFP-C1 or EGFP-Parkin. After 24 h, the cells were induced with 100 μΜ H_2_O_2_ for 0 h or 12 h and immunofluorescently stained with a p-GSK3β-S9 (red) antibody. The arrows show the aggregation of p-GSK3β-S9 in the nucleus. Scale bar, 20 μm.

**Supplementary Figure 9. Parkin-mediated mitophagy delays H_2_O_2_-induced cell death.**

Cell viability was detected after H_2_O_2_ induction (100 μΜ; 0 h, 18 h or 24 h) in stable cell lines (EGFP-Control and EGFP-Parkin). N = 3; ns, not significant; *P < 0.05; ***P <0.001. Data from three independent tests were collected for statistical analysis (mean ± SD).

**Supplementary Figure 10. Changes in mitophagy-associated protein expression and mitochondrial morphology in *OPTN-KO* and miR-25-KO HeLa cells.**

**(A, C)** The levels of MFN2, OPTN, NDP52 and TOMM40 were evaluated by WB analysis in WT and *OPTN-KO* HeLa cells.

**(B)** The levels of MCU in WT cells and three miR-25-deleted cell lines were evaluated by WB analysis. N = 3; **P < 0.01; ***P <0.001. The data are from three independent tests and are presented as the mean ± SD.

**(D)** Ratios of related proteins to Tubulin in *OPTN-KO* cell lines. N = 3; ns, not significant; **P < 0.01; ***P <0.001. Data from three independent tests were collected for statistical analysis (mean ± SD).

**(E)** Mitochondria in WT and *OPTN-KO* HeLa cells were stained with MitoTracker Red (red) to label the mitochondrial morphology. Scale bar, 20 μm.

**(F)** WT and *OPTN-KO* HeLa cells were transfected with EGFP-Parkin and then stained with MitoTracker Red (red) to label the mitochondrial morphology. Scale bar, 10 μm.
