# Supplementary material for: Oxidative stress-induced mitophagy is suppressed by the miR-106b-93-25 cluster in a protective manner
Source: Cell Death Dis. 2021 Feb 24;12(2):209. doi: 10.1038/s41419-021-03484-3 (PMC7904769; doi:10.1038/s41419-021-03484-3)
Supplement: Supplementary file 13 — Supplementary Tables [file 41419_2021_3484_MOESM13_ESM.docx]

**Zhang, C. et al_Supplementary Tables**

**Supplementary Table 1. Sequences of oligos for miR-106b, miR-93 and miR-25 gene KO**

| **Name** | **Sequence (5’-3’)** |
| --- | --- |
| **106b-1** | ACCGTGGGTACTTGCTGCTCCAGC |
| **106b-2** | AAACGCTGGAGCAGCAAGTACCCA |
| **93-a** | ACCGAGCTAGCACTTCCCGAGCCCC |
| **93-b** | AAACGGGGCTCGGGAAGTGCTAGCT |
| **25-a** | ACCGCCCTGGGCATTGCACTTGTCT |
| **25-b** | AAACAGACAAGTGCAATGCCCAGGG |

**Supplementary Table 2. List of primers used for construction of shRNA plasmids**

| **Name** | **Sequence (5’-3’)** |
| --- | --- |
| **NRF2-1** | CCGGCCGGCATTTCACTAAACACAACTCGAGTTGTGTTTAGTGAAATGCCGGTTTTTG |
| **NRF2-2** | CCGGGCTCCTACTGTGATGTGAAATCTCGAGATTTCACATCACAGTAGGAGCTTTTTG |
| **shCtrol** | CCGGCCTAAGGTTAAGTCGCCCTCGCTCGAGCGAGGGCGACTTAACCTTAGGTTTTTG |

**Supplementary Table 3. List of LNA-labeled primers (the bold bases were labeled with LNA, and the 3' ends were labeled with DIG)**

| **Name** | **Sequence (5’-3’)** |
| --- | --- |
| **106b-dig-LNA** | 5-ATCT**G**CACT**G**TCAG**C**ACTTTA-3 |
| **93-dig-LNA** | 5-CTACCTG**C**ACGAA**C**AGCA**C**TTTG-3 |
| **25-dig-LNA** | 5-TCAGA**C**CGAGA**C**AAGT**G**CAATG-3 |
| **U6-dig-LNA** | 5-GCAGGG**G**CCATG**C**TAAT**C**TTCTCTGT-3 |

**Supplementary Table 4. List of stem-loop primers for the RT reaction**

| **Name** | **Sequence (5’-3’)** |
| --- | --- |
| **106b-loop** | GTCGTATCCAGTGCAGGGTCCGAGGTATTCGCACTGGATACGACATCTGCAC |
| **93-loop** | GTCGTATCCAGTGCAGGGTCCGAGGTATTCGCACTGGATACGACCTACCTGC |
| **25-loop** | GTCGTATCCAGTCGAGGGTCCGAGGTATTCCGACTGGATACGACTCAGACCG |
| **U6rt (as control)** | CCTTGCGCAGGGGCCATGCTAATC |

**Supplementary Table 5. List of RT-qPCR primers for miRNAs and related gene detection**

| **Name** | **Sequence (5’-3’)** |
| --- | --- |
| **106bjc1:** | CGCGGTAAAGTGCTGACAGTG |
| **106bjc2:** | CAGTGCAGGGTCCGAGGTAT |
| **93j c1** | CGCGGCAAAGTGCTGTTC |
| **93jc2** | CAGTGCAGGGTCCGAGGTAT |
| **25jc1** | GGCGGCATTGCACTTGTCTCG |
| **25jc2** | GTCGAGGGTCCGAGGTATTCCG |
| **U6P1 (as control)** | TCCAATTTTAGTATATGTGC |
| **U6P2 (as control)** | CGATACAGAGAAGATTAGC |
| **OPTN q-F** | GCTCATACAAAACTCAGCGAA |
| **OPTN q-R** | TTTCATCCTCTGTTTTAGCCT |
| **MFN2 q-F** | ATAGATGGCTTGAAACCCCT |
| **MFN2 q-R** | GCCCAGGAACCTATTCACC |
| **NDP52 q-F** | GACAACCCGTGAGTATTACACC |
| **NDP52 q-R** | TGGAAAGGAATACTTGCTCCC |
| **p62 q-F** | CCCACGGCAGAATCAGCTT |
| **p62 q-R** | GCTTCTTTTCCCTCCGTGCTC |
| **MFN1 q-F** | AAAATCAAGGAGGTTACCGA |
| **MFN1 q-R** | ACTAAGGCGTTTACTTCATCG |
| **Tubulin q-F (as control)** | CGAAGCCAGCAGTGTCTAAA |
| **Tubulin q-R (as control)** | GGAGGACGAGGCCATAAATA |

**Supplementary Table 6. List of PCR primers for amplification of the 3’UTRs of the human *OPTN, MFN2* and *NDP52/CALCOCO2* genes.**

| **Name** | **Sequence (5’-3’)** |
| --- | --- |
| **OPTN-UTR-F** | CACTGGTCCTCGAGGTGTTGATGTATCACCTCCCCA |
| **OPTN-UTR-R** | ATCAAGGAATGCGGCCGCTCGTCTCGGCTCACTGCAAC |
| **MFN2-UTR-F** | CACTGGTCCTCGAGGTCAGGGAAAATCACTGTCA |
| **MFN2-UTR-R** | ATCAAGGAATGCGGCCGCGAGCAATGATACAGGAGTAAC |
| **NDP52-UTR-F** | CACTGGTCCTCGAGCCAGAGATTGGTCCGAGGTTG |
| **NDP52-UTR-R** | ATCAAGGAATGCGGCCGCTTGGACGGCTGAAGTGGACA |
